# Comparative phylogeography uncovers evolutionary past of Holarctic dragonflies

**DOI:** 10.7717/peerj.11338

**Published:** 2021-06-24

**Authors:** Manpreet Kohli, Marie Djernæs, Melissa Sanchez Herrera, Göran Sahlen, Erik Pilgrim, Thomas J. Simonsen, Kent Olsen, Jessica Ware

**Affiliations:** 1 Department of Invertebrate Zoology, American Museum of Natural History, New York, New York, United States; 2 Department of Biological Sciences, Rutgers, The State University of New Jersey, Newark, New Jersey, United States; 3 Natural History Museum Aarhus, Aarhus, Denmark; 4 Faculty of Natural Sciences, Universidad del Rosario, Bogotá D.C., Colombia; 5 The Rydberg Laboratory for Applied Sciences, Halmstad University, Halmstad, Sweden; 6 Department of Biology, Utah State University, Logan, Utah, United States; 7 Department of Biology, Aarhus University, Aarhus, Denmark

**Keywords:** Dragonflies, Phylogeography, Holarctic, Aeshna, Somatochlora, Circumboreal, Sympetrum, Libellula, Beringia

## Abstract

Here, we investigate the evolutionary history of five northern dragonfly species to evaluate what role the last glaciation period may have played in their current distributions. We look at the population structure and estimate divergence times for populations of the following species: *Aeshna juncea* (Linnaeus), *Aeshna subarctica* Walker, *Sympetrum danae* (Sulzer), *Libellula quadrimaculata* Linnaeus and *Somatochlora sahlbergi* Trybom across their Holarctic range. Our results suggest a common phylogeographic pattern across all species except for *S. sahlbergi*. First, we find that North American and European populations are genetically distinct and have perhaps been separated for more than 400,000 years. Second, our data suggests that, based on genetics, populations from the Greater Beringian region (Beringia, Japan and China) have haplotypes that cluster with North America or Europe depending on the species rather than having a shared geographic affinity. This is perhaps a result of fluctuating sea levels and ice sheet coverage during the Quaternary period that influenced dispersal routes and refugia. Indeed, glacial Beringia may have been as much a transit zone as a refugia for dragonflies. *Somatochlora sahlbergi* shows no genetic variation across its range and therefore does not share the geographic patterns found in the other circumboreal dragonflies studied here. Lastly, we discuss the taxonomic status of *Sympetrum danae*, which our results indicate is a species complex comprising two species, one found in Eurasia through Beringia, and the other in North America east and south of Beringia. Through this study we present a shared history among different species from different families of dragonflies, which are influenced by the climatic fluctuations of the past.

## Introduction

Northern habitats provide a unique opportunity for studying evolutionary processes because of their recent geographic and climatic past and imminent threat of current climate change (Strathdee et al., 1995). Glaciation cycles of the Quaternary period (~2.6 mya) have influenced the current biodiversity, distribution patterns and genetic diversity of northern Holarctic taxa (Hewitt, 2004; Hewitt, 2000). During the Quaternary period, as glacial covers increased, species went extinct, moved south or survived in local refugia (e.g., insects (Bidegaray-Batista et al., 2016; García-Vázquez et al., 2017), plants, arachnids (Wachter et al., 2016), mammals (Polfus et al., 2017)). While range expansions may have occurred in cold adapted species, temperate species, on the contrary, might have experienced range contractions through evolutionary time (Tzedakis, Emerson & Hewitt, 2013). As post-glaciation temperatures increased and habitats were recolonized (Crawford, 2013; Theissinger et al., 2013; Theissinger et al., 2011), cold adapted species may have faced habitat change and/or new competition. Currently, we see rapid changes to global climate and habitats, similar to or worse than those following global warming after the last glacial maximum (McInerney & Wing, 2011). These climatic changes are actively shaping ecological communities (Parmesan & Yohe, 2003) and we are racing against time to document and understand community response, vital for conservation. One way to do this is to use past responses to climate change as predictors of future response.

Phylogeographic studies that focus on species during the Quaternary period, when significant climatic variations took place could help us determine: (1) how changes in dispersal or migration patterns may influence range expansion and contraction, (2) migratory routes and how taxa overcome barriers to migration and (3) which regions have acted as refugia during glaciation cycles. Several studies have investigated how the glaciation cycles have influenced the distribution and evolutionary history of organisms, but these have generally concentrated on mammals and birds. A handful of studies have considered freshwater invertebrates, but these focused on taxa with limited geographic ranges; usually studying the response of taxa living in the mountains of northern Europe (Theissinger et al., 2013; Theissinger et al., 2011; Lehrian, Pauls & Haase, 2009; Lehrian et al., 2010; Pauls et al., 2013; Pauls et al., 2009; Simonsen, Olsen & Djernæs, 2020). However, studying species with expansive ranges across the Holarctic provides a unique opportunity to understand the effect of the recent glaciation cycles on intraspecific genetic diversity across continents.

Here, we study one particular group of freshwater invertebrates, Odonata (dragonflies and damselflies), which are vagile, highly mobile taxa with terrestrial adults and aquatic larvae/nymphs. We particularly focus on odonate species that have a circumboreal range. Only five dragonfly species, *Aeshna juncea* (Linnaeus), *A. subarctica* Walker, *Somatochlora sahlbergi* Trybom, *Libellula quadrimaculata* Linnaeus and *Sympetrum danae* (Sulzer), are known to have both Palearctic and Nearctic distributions with populations existing north of the arctic circle (Corbet, 1999; Cannings & Cannings, 1997). Apart from *S. sahlbergi* and *L. quadrimaculata*, none of these species have been treated in a phylogeographic context before. Kohli et al. (2018) studied *S. sahlbergi* across its circumboreal range and found a surprising pattern: there is no haplotypic diversity in the mitochondrial gene Cytochrome Oxidase I (COI) and hence no genetic structure among populations of this species across the continents of North America and Europe. This is perhaps because of any one of three possible scenarios: (1) *S. sahlbergi* populations were connected until very recently, till the end of the Last Glacial Maxima (LGM) (~11,000 years ago), and/or (2) *S. sahlbergi* has a longer generation time than most odonates leading to slow accumulation of genetic changes, and/or (3) this species has spread quickly from a small and homogenous refugial population recently. Virtually nothing is known about the natural history of *S. sahlbergi* due to its remote habitat, therefore, it is difficult to come to a definitive explanation for these patterns. By contrast, Artiss (2004) studied the phylogeography of *L. quadrimaculata* and concluded that there was genetic structure across its range; there are three distinct populations in Japan, North America and Europe. However, from this analysis which was based on a handful of specimens, the structure and relationships between these populations were unclear.

In this manuscript, we study the phylogeography of *A. juncea, A. subarctica, S. sahlbergi*, *L. quardrimaculata* and *S. danae*. We investigate whether these five species which belong to three different families (Aeshnidae, Corduliidae and Libellulidae) show geographic structure across their large Holarctic range similar to those reported by Artiss (2004) in *L. quadrimaculata*. Or, instead, if there are no genetic differences in these species across the continents, like *S. sahlbergi* (Kohli et al., 2018). Ultimately, we compare and contrast the evolutionary patterns in these species and address several questions. Did these five dragonfly species, which currently have similar continental distributions, share an evolutionary path? Did the Quaternary glaciation cycles affect each species in a similar way? Such questions help us understand the factors that guide the speciation process at the higher latitudes. This is particularly interesting in the context of topography, given that mountain ranges may have structured glacial advances differently in North America (where mountain ranges limit east-west glacial movement) and Eurasia (where mountains limit north-south glacial movement), which in turn likely shaped dragonfly movement.

## Methods

### Taxon sampling and dataset preparation

Our taxon sampling strategy included multiple locations in North America and Europe (Table S1). Overall, we collected a total of 109 individuals for the following species: *A. juncea* (*n* = 56), *A. subarctica* (*n* = 18), *L. quadrimaculata* (*n* = 14) and *S. danae* (*n* = 21). To augment our taxon sampling for these four species, we downloaded COI sequences publicly available on NCBI Genbank and the Barcode of Life Database (BOLD) (Table S1). This increased our total sampling for *A. juncea* to 106, *A. subarctica* to 33, *L. quadrimaculata* to 60 and *S. danae* to 159 individuals. In addition, *Somatochlora sahlbergi* samples (*n* = 48) used by Kohli et al. (2018) were included in our study.

### DNA extraction and amplification

For the samples collected as part of this study (Table S1) DNA extractions were performed either at Rutgers University-Newark, New Jersey (NJ), USA, Utah State University, Logan, Utah (UT), USA or at Aarhus University, Denmark. For all samples extracted in NJ and UT, we followed the QIAGEN DNeasy Tissue Kit manufacturer protocol, but with an extended incubation time of 24 h to increase DNA yield. For all samples extracted in Denmark, we followed the E.Z.N.A. Tissue DNA Kit manufacturer protocol, but with an extended lysis incubation time (+18 h) and a lower temperature (37–42 °C), skipping steps 5 and 6 (centrifugation and transfer to a new tube after lysis), and incubating at 70 °C for +5 min with elution buffer.

We amplified the animal barcode gene *Cytochrome Oxidase subunit 1*, a fast-evolving mitochondrial gene, demonstrated to show variation at the population and subpopulation level in insects (Theissinger et al., 2013; Dinsdale et al., 2010; Lim et al., 2012; Simonsen & Huemer, 2014; Troast et al., 2016). For all samples extracted in NJ, we followed the PCR protocol previously detailed in Kohli et al. (2014) using primers listed in Table S2. All PCR products were sequenced using Sanger Method in the Macrogen New York sequencing facilities or at UT. Contigs and consensus sequences were obtained using sequencher v. 5.4.6 (Gene Codes Corporation, Ann Arbor, MI USA). For all samples extracted in Denmark, we used the following PCR protocol: 95 °C, 2 min; then 35–45 cycles at 95 °C, 30 s; 45 °C, 30 s; 72 °C, 1 min and a final extension of 72 °C for 5 min using the primers listed in Table S2. All PCR products were sequenced using the Sanger Method in the Macrogen Europe sequencing facilities. Contigs and consensus sequences were obtained using DNA Baser Sequence Assembler v. 5.8.0 (Heracle BioSoft, 2018). Finally, we generated separate sequence alignments for each of the species, using ClustalX (Larkin et al., 2007) and then manually checked for incongruencies in Mesquite (Maddison & Maddison, 2019). The final alignment length for all the species was 657 bp except for *S. danae*, which was 990 bp. All the newly generated sequences were deposited in the NCBI GenBank database (Table S1 for accession numbers). All the final alignments are available upon request from the corresponding author.

### Population genetics analysis

To explore the genetic diversity for each target species we estimated and visualized relationships between haplotypes within a species using Minimum Spanning Networks (Bandelt, Forster & Rohl, 1999) as implemented in the population genetic software package PopART (Leigh & Bryant, 2015). In addition, we estimated the following molecular polymorphism statistics for each species: number of haplotypes (h), segregation sites (S), and genetic diversity (π) using the population genetics suite Arlequin v. 3.5.2.2 (Excoffier, Laval & Schneider, 2005), by country of origin of the sampling.

In order to establish the number of genetic clusters/populations (K) within each species, we used the K-means clustering analysis with the *adegenet* R package (Jombart & Ahmed, 2011; Jombart, 2008). The optimal clustering solution was selected by looking at the lowest Bayesian Information Criterion (BIC). In practice, the ‘best’ BIC is often indicated by an elbow in the curve of BIC values as a function of K clusters. We used the Discriminant Analysis of Principal Components (DAPC) method as implemented in *adegenet* R package to explore the genetic variation among the identified population clusters for each of the species. Moreover, we determined the membership probability of each sample for each of the predicted populations across all five targeted species. Finally, we calculated the degree of genetic differentiation F_ST_ among the predicted clusters for all species using *hierfstat* R package, and we ran an AMOVA in the *poppr* R package (Kamvar, Brooks & Grünwald, 2015; Kamvar, Tabima & Grünwald, 2014) to test whether the observed variation was higher among or within each cluster.

### Divergence time estimation analysis

We performed divergence time estimation analysis in the software package beast v. 2.5.2 (Bouckaert et al., 2014). As no fossil record exists for any of the five species studied here, we had to rely on secondary calibrations as priors for the divergence time analyses. To establish these secondary calibrations, we first estimated the fossil calibrated age of each of the species within each genera *Aeshna*, *Somatochlora*, *Sympetrum* and *Libellula* using divergence time estimation methodology as implemented in beast v. 2.5.2. Finally, time posteriors obtained from these analyses were used as priors for conducting a subsequent divergence time analysis to estimate the timing of split between various populations within each species. Results from divergence time estimation analysis were presented as Chronograms, i.e., phylogenies scaled with time. Details of the samples, alignments, models of evolution, fossils and fossil priors are provided in Appendix S1 and Tables S3.1–S3.3.

## Results

### Haplotype diversity

We recovered high haplotype diversity in some species (e.g. *A. juncea* and *L. quadrimaculata*) while others showed only very little diversity (e.g. *S. sahlbergi*). To understand these results in a larger geographical context, we combined samples from European countries into one geographic region (Europe) and Canada and the United States into another region (North America). However, Alaska and Yukon Territory (hereafter referred to simply as “Yukon”) were an exception to this, and together they were treated as a separate region. This is because our analysis indicates that these two areas show a genetic variation different from other North American areas. For similar reasons Russia is discussed separately from Europe, and Japan and China are treated separately instead of treating them together as Asia.

**Figure 1:**
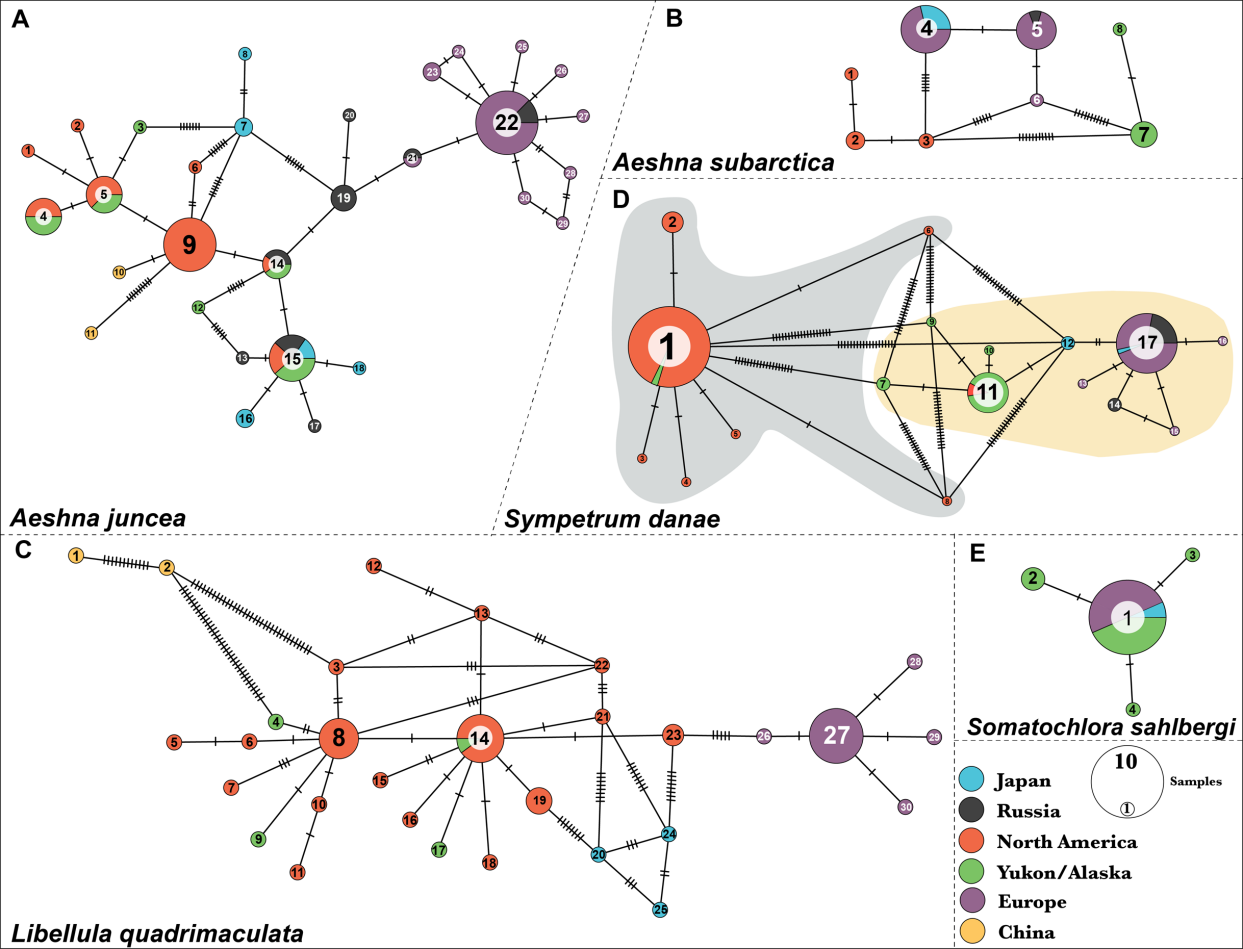
Haplotype networks for the five dragonfly species. (A) *A. juncea*, (B) *A. subartica*, (C) *L. quadrimaculata*, (D) *S.danae*, and (E) *S. sahlbergi*. Each circle represents a unique haplotye and the size of the circle is proportional to the number of the samples that share that haplotype. Haplotypes are connected to each other by lines and the ticks on the connecting lines indiacate number of nucleotide changes between the haplotypes. Each haplotype (circle) is colored according the geographic affinity of the samples within it. *All the haplotypes within each network are numbered*. Two very divergent groups were recovered in *S. danae*. Group A is highlighted in gray color while Group B is highlighted in yellow color.

***Aeshna juncea***—We identified thirty different haplotypes for *A. juncea*. The minimum spanning network shows the relationships between the haplotypes (Fig. 1A). Results show that North American and European individuals do not share any haplotypes. Individuals from North America show eight different haplotypes and most of them share haplotype #9. In samples from Yukon–Alaska six haplotypes were recovered, out of which four are shared with North American samples. Europe showed two more haplotypes compared to North America; however, most are just one or two nucleotides different from the most common haplotype, #22 (Fig. 1A).

Results show five haplotypes among individuals from Japan. One of these haplotypes, #15, is shared with North American samples. Two haplotypes, #16 and #18, are closely related to North America, while the remaining two, #6 and #7, are closely related to Europe (Fig. 1A). In fact, Japanese individuals are 2.9% genetically different from each other (Table S4). The two individuals from China were recovered as two separate haplotypes and both are connected to haplotype #9, indicating that they are closely related to populations from North America. These two individuals show an unusual amount of genetic diversity, with 16 nucleotide differences between them. Lastly, out of the total eight haplotypes identified from Russia, haplotypes #14 and #15 are shared with North American individuals while haplotypes #21 and #22 are shared with European samples. On an average, Russian individuals are 2.01% genetically different from each other (Table S4).

***Aeshna subarctica***—We identified eight haplotypes for *A. subarctica* (Fig. 1B). All the European individuals fall in three different haplotypes, #4, #5 and #6. However, most of them share haplotype #4 and #5, which in turn are separated by one nucleotide difference. In North American samples, three different haplotypes, #1, #2, and #3, were recovered; while, two very divergent haplotypes, #7 and #8 were recovered in the Yukon–Alaska samples (Fig. 1B). Additionally, Japan and Russia in this case share haplotypes with Europe. Individuals from Japan exhibit haplotype #4 while, Russian samples exhibit haplotype #5 (Fig. 1B).

***Somatochlora sahlbergi***—We identified four haplotypes for *S. sahlbergi* (Fig. 1E) in agreement with Kohli et al. (2018). The majority of individuals collected across North America and Europe share one haplotype, #1. The remaining three haplotypes differ from haplotype #1 by only one nucleotide difference. Detailed discussions of this haplotype structure and about *S. sahlbergi* can be found in Kohli et al. (2018).

***Libellula quadrimaculata***—We identified thirty haplotypes for *L. quadrimaculata* (Fig. 1C). The haplotype network shows that most of the variation is present in samples from North America (seventeen haplotypes) compared to European samples (five haplotypes) (Fig. 1C). Most of the European individuals share haplotype #27, with four haplotypes connected to it that differs by only one nucleotide difference (Fig. 1C). On the other hand, North American diversity revolves around the two main haplotypes #8 and #14, and several other single individual haplotypes that are connected to those main ones, that differ by one, two or three nucleotides (Fig. 1C). The two Chinese samples seem to be very divergent from the North American samples with a 2.5% average pairwise difference among them. Further, these two Chinese samples show a pairwise difference of 1.8% between them. All mean polymorphism statistics for this species show a genetic diversity comparable with *A. juncea* (see Table S4).

***Sympetrum danae***—We identified seventeen different haplotypes for *S. danae*, where most of the samples share either of the following haplotypes: #1, #11 or #17 (Fig. 1D). Our haplotype network shows that haplotype #1 is quite divergent from #11 and #17, showing a high number of point mutations among them (Fig. 1D). In terms of geographic affinities, we find that most of the North American individuals, collected across Canada and the United States share haplotype #1, while most individuals from Yukon–Alaska exhibit #11 and related haplotypes. All European, including Russian, individuals exhibit haplotype #17 and other closely related haplotypes. Two Japanese samples were grouped in one haplotype, #9, which is closer to the Yukon–Alaska haplotype (#11). One remaining Japanese sample shares haplotype #17 with all European samples. From here on we refer to haplotype #1 and its connected haplotypes as *S. danae* group A (Grey color in Fig. 1D), while we refer to #11 and #17 and connected haplotypes as *S. danae* group B (Yellow color in Fig. 1D). These two groups show a genetic divergence of 6.67%. Overall, group B (i.e. Europe, Russia, Japan, Yukon–Alaska) seems to show a higher mean nucleotide diversity in comparison than group A (North America, few Yukon–Alaska) (Table S4).

### Population clusters, F_ST_ and AMOVA

In the following section we report results from the DAPC analysis using the k-means clustering algorithm, including the recovered populations and genetic mitochondrial differentiation between these populations estimated through F_st_ and AMOVA.

**Figure 2:**
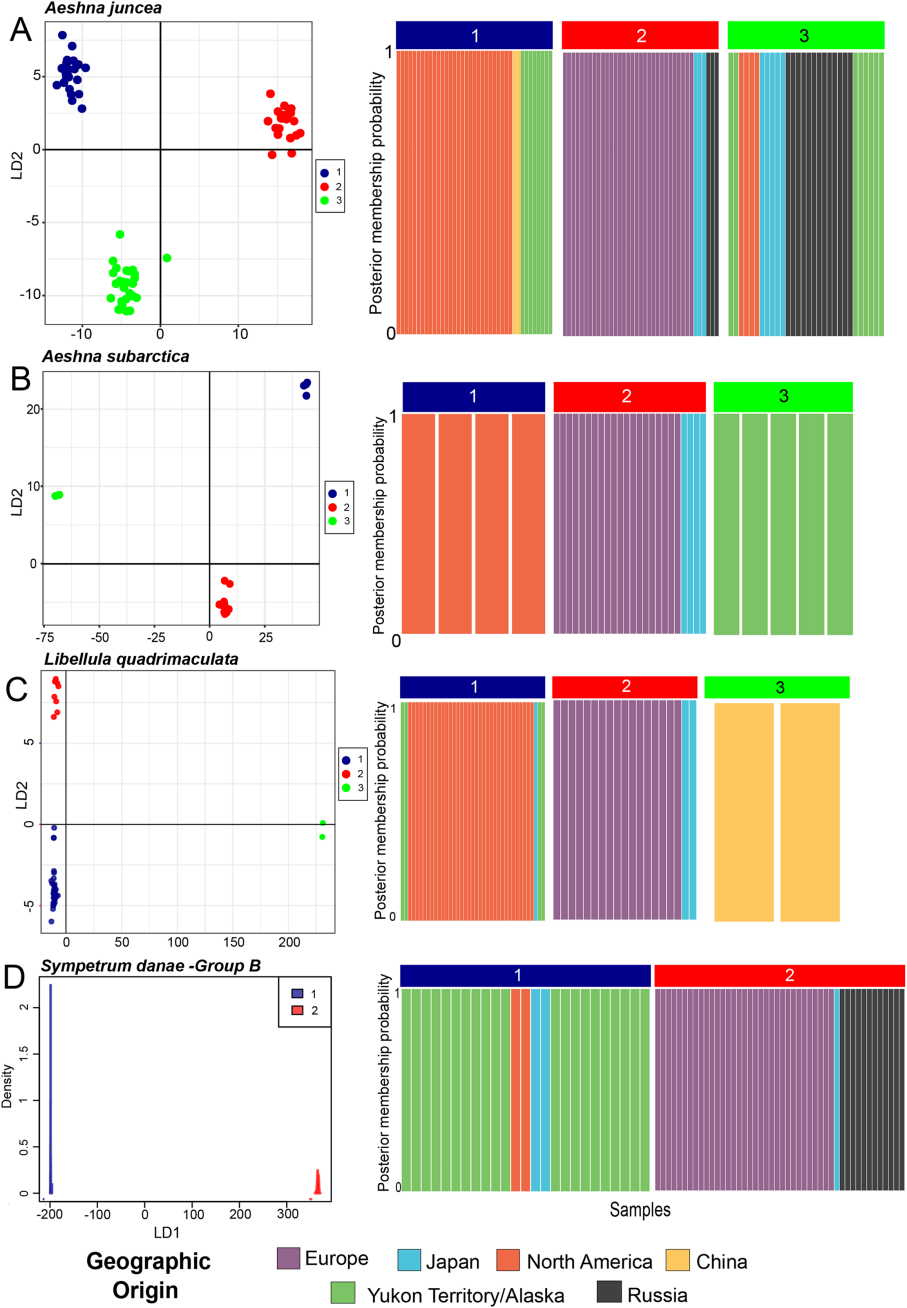
Predicted population cluster and DAPC analyses. For all species we show the Linear Discriminant scatterplot on the left and the membership probability plots on the right. (A) *Aeshna juncea*, (B) *Aeshna subartica*, (C) *Libellula quadrimaculata* and (D) *Sympetrum danae*-group B. All the linear discriminant plots show separation of the populations along Linear Discriminant axis 1 (LD1) and 2 (LD2), except for *S. danae*, since there are only two populations that are separated only on LD1. In the membership probability plot all individuals are colored according to their geographical origin.

***Aeshna juncea***—Using the DAPC analysis we determined three (K = 3, Fig. S1) to be the appropriate numbers of genetic clusters for *A. juncea*. Cluster #1 primarily comprises all individuals from North America, two from China and eight from Yukon and Alaska (Fig. 2A). Cluster #2 comprises all the European samples with some Japanese and Russian individuals (Fig. 2A). Lastly, cluster #3 comprises an admixture of samples from Russia, Japan, Yukon–Alaska, as well as a few other North America samples. The pairwise F_ST_ estimate among these three genetic clusters show high genetic differentiation between the North America and European clusters (Table 1). Although, there is a low genetic differentiation between the North America and mix cluster #3, suggesting possible gene flow (see Table 1). AMOVA values indicate that 74% of the genetic mitochondrial variation is explained by differences between the populations, and only 26% is explained by differences within populations.

**Table 1:** Estimated Pairwise F_ST_ values for the predicted genetic clusters for each of the target dragonfly species.

*Species*	Cluster	#1	#2	#3
*Aeshna juncea*	**#1**	0.00	0.79	0.28
**K = 3**	**#2**	0.79	0.00	0.65
	**#3**	0.28	0.65	0.00
				
*Aeshna subarctica*	**#1**	0.00	0.66	0.89
**K = 3**	**#2**	0.66	0.00	0.84
	**#3**	0.89	0.84	0.00
				
*Sympetrum danae* (Group B)	**#1**	0.00	0.48	
**K = 2**	**#2**	0.48	0.00	
				
*Libellula quadrimaculata*	**#1**	0.00	0.50	0.73
**K = 3**	**#2**	0.50	0.00	0.48
	**#3**	0.73	0.48	0.00

***Aeshna subarctica***—Using the DAPC analysis we determined three (K = 3, Fig. S1) to be the appropriate numbers of genetic clusters for *A. subarctica*, despite the low taxon sampling (Fig. 2B). Cluster #1 groups all North American samples, while cluster #2 groups all European and Japanese samples. Finally, cluster #3 comprises all the specimens from Yukon and Alaska. Pairwise F_ST_ estimate among the three predicted genetic clusters show high or intermediate genetic differentiation (Table 1). AMOVA values indicate that 91% of the genetic mitochondrial variation is explained by differences between the populations, while only a 9% is explained by differences within populations.

***Somatochlora sahlbergi***—Using the DAPC analysis we were not able to identify an appropriate number of clusters. All the clusters garnered negative BIC scores indicating that there is no appropriate number of clusters in this population (Fig. S1).

***Libellula quadrimaculata***—Using the DAPC analysis we determined three (K = 3, Fig. S1) to be the appropriate numbers of genetic clusters for *L. quadrimaculata* (Fig. 2C). Cluster #1 comprise North America including Yukon and Alaska, cluster #2 groups mostly European samples, and finally cluster #3 is composed only of the highly divergent Chinese samples (Fig. 2C). Japan shows samples in two clusters, #1 and #2, similarly to the other species (Fig. 2C). Our F_ST_ estimates among the predicted clusters show that there is moderate genetic differentiation (Table 1). The AMOVA results show that around 79% of the genetic mitochondrial variation is explained by differences between the populations, while 20% is explained by within population differences.

***Sympetrum danae***—Using DAPC analysis we confirmed that the *S. danae* A and *S. danae* B groups are very divergent with the pairwise F_ST_ = 0.93 between the two clusters indicating almost complete differentiation between them (Fig. S2). These results are consistent with results of haplotypes network analysis for this species (Fig. 1D). In the rest of the manuscript we only discuss results from *S. danae* group B, because this group has a circumboreal distribution, and *S. danae* group A is restricted to North America (including Yukon–Alaska), shows very little variation and no geographical pattern. Using DAPC analysis we recovered two well defined genetic clusters for taxa in the *S. danae* group B (Fig. 2D). The density plot for the linear discriminant shows that there is some variation within the predicted populations (Fig. 2D). Samples from Yukon and Alaska are grouped with a couple of individuals from North America (Cluster #1), while the other group is composed by European samples including Russia (Cluster #2, Fig. 2D). For *S. danae*, Japan shows individuals in both genetic clusters. The F_ST_ estimate was 0.47, suggesting some genetic differentiation between the Yukon–Alaska and European populations. Our AMOVA results suggest that around a 74.5% of the genetic variation observed is between the populations, while 25.5% is explained by within population differences.

### Divergence time estimates

In this section we present the results from the divergence time estimation analysis for all the species.

**Figure 3:**
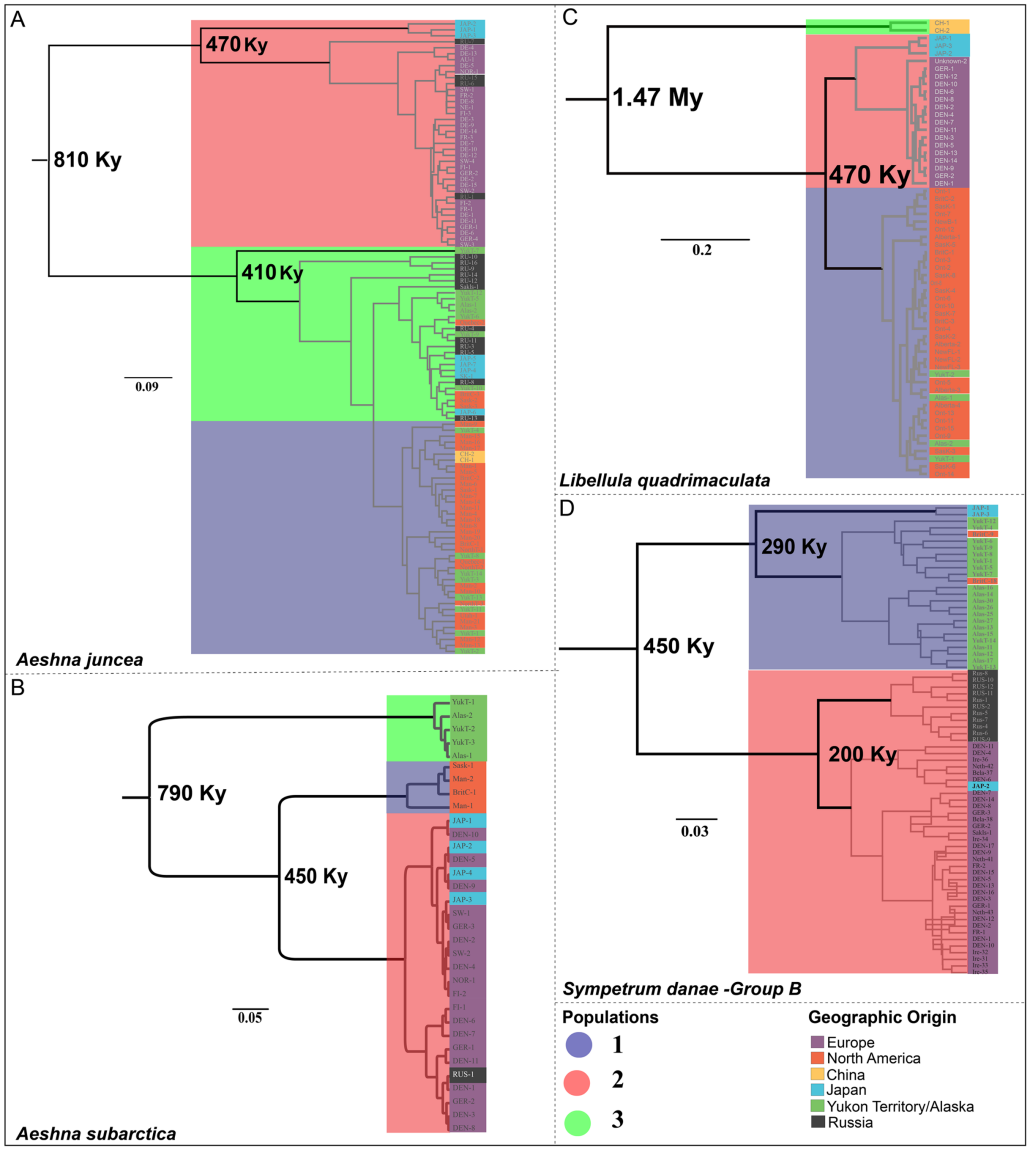
Chronograms showing the divergence time estimates for the four Holarctic dragonflies. Mean estimated ages for the nodes are indicated in bold. Major clades are highlighted in accordance with predicted population clusters as shown in Fig. 2. Each sample within a population is colored according to its geographic affinity.

***Aeshna juncea***—The three populations #1, #2 and #3 (Fig. 2A) are recovered in two different clades in the divergence time estimation analysis (Fig. 3A). Clade 1 is comprised by individuals from Europe, Japan and Russia, as in Cluster #2 (Fig. 3A). Clade 2 is comprised of populations #1 and #3, which are overall comprised by individuals form North America, Yukon–Alaska, and some individuals from Russia and Japan. The results indicate that the two clades, and therefore, the European and the North American populations of *A. juncea* split *ca* 810 Kya (95% confidence interval range: 220 Kya–2.9 Mya). The prior for this analysis was set at 220 Kya–6.9 Mya (Table S3.3) indicating that data are informative and influencing the posterior estimates that are recovered. The most recent common ancestor (MRCA) for population #2, which predominantly consists of individuals from Europe, existed 470 Kya (95% confidence interval range: 30 Kya–1.7 Mya), while the MRCA for populations #1 and #3 was recovered to be 410 Ky old (95% confidence interval range: 20 Kya–1.5 Mya).

***Aeshna subarctica***—The MRCA for all *A. subarctica* populations existed ca. 790 Kya (95% confidence interval range: 220 Kya–2.82 Mya) (Fig. 3B). We specified the prior for this split as 220 Kya–6.9 Mya (Table S3.3), indicating that our data was informative and influenced the posterior shape. Population #3 (Fig. 2B), which contains individuals from Yukon and Alaska is recovered as a sister to the rest of the specimens, which are divided into two populations. The split between population #1 (North America) and population #2 (predominantly Europe) is estimated to have happened around 450 Kya (95% confidence interval range: 45.8 Kya–1.6 Mya) (Fig. 3B).

***Somatochlora sahlbergi***—None of the diversity estimates for *S. sahlbergi* were meaningful because of an absence of molecular diversity in the COI (and D2, see Kohli et al. (2018)) shown by this species. According to our divergence time estimates S. *sahlbergi* diverged from its sister species, *Somatochlora alpestris* around 1.87 Mya.

***Libelulla quadrimaculata***—The MRCA for all populations of *L. quadrimaculata* existed ca. 1.47 Mya (95% confidence interval range: 570 Kya–3.9 Mya) (Fig. 3C). Two genetically divergent individuals from China (Figs. 1C and 2C) are recovered as sister to rest of the populations. The predominantly North American population #1 split from population #2 (European and Japanese individuals) ca. 470 Kya (95% confidence interval range: 60 Kya–1.35 Mya).

***Sympetrum danae***—The MRCA for all populations of *S. danae* sensu lato existed ca. 1.7 Mya (95% confidence interval range: 120 Kya–4.1 Mya) when Group A in North America split from the rest of the *S. danae* populations (Fig. 3D). The MRCA for Group B was recovered to be *ca* 450 Ky old (95% confidence interval range: 12 Kya–1.6 Mya). Population #1, primarily Yukon–Alaska, was recovered to be *~*290 Ky old (95% confidence interval range: 18 Kya–3.9 Mya), while population #2 (Europe) was recovered to be *ca*. 200 Ky old (95% confidence interval range: 12 Kya–3.4 Mya).

## Discussion

This is the first empirical comparative phylogenetic assessment of Holarctic dragonflies. Overall, our results highlight the following interesting comparative patterns among these five dragonfly species: the “Atlantic divide” and the “muddle in the middle”.

### The Atlantic divide: North American and European populations

The first pattern we find is the presence of genetic differences between populations from North America and Europe. Individuals in these continental populations have separate haplotypes across their circumboreal range and there is not a single instance of a shared haplotype between North American (including Yukon and Alaska) and European samples (Figs. 1, 2). A similar observation was recently made by Galimberti et al. (2020) for *A. juncea* and *S. danae*. In addition, we recover high to moderate F_ST_ estimates (Table 1) between these populations suggesting that there is almost no gene flow between them leading to geographic structuring. This perhaps indicates that these continental populations have been separated for some time. The only exception to this pattern is *S. sahlbergi*, which is discussed below. If the non-*Somatochlora* populations have strong genetic structure, then, when and where did these populations separate?

The continents of North America and Eurasia were created upon the breakup of the supercontinent Laurasia. This break started about 200 Mya and full separation of the two continents was completed around 55 Mya (Pedersen et al., 2012). However, all the species that we have considered here are unlikely to be that old and therefore vicariance (that is, splitting of Laurasia) does not explain the genetic separation between North American and European populations. Indeed, our divergence time estimates suggests all these species are relatively young and that the split between North American and European populations (Fig. 3) for all the species occurred within the last ~800,000 years. In fact, except for *A. juncea*, the splits between European and North American populations of *A. subarctica*, *S. danae*, and *L. quadrimaculata* are dated to be ~450,000 years ago. Because we are using only one molecular marker for our analysis there is a large amount of uncertainty associated with our data, which explains the large confidence intervals around our estimates (Rannala & Yang, 2007; Yang & Rannala, 2006). However, the upper limit of the confidence intervals for the basal splits in *A. subarctica*, *L. quadrimaculata* and *S. danae* were well within the last two million years, much younger than the vicariant event of the Laurasia split. For *A. juncea*, which was estimated to be older, the upper age limit estimate was similarly too young (~2.9 Mya) to suggest vicariance explanations. Could it be that these taxa diverged on one continent, dispersed to the other continent, and then due to environmental factors continued dispersal was lessened? For the four taxa (*A. juncea, A. subarctica*, *S. danae*, and *L. quadrimaculata*) the divergence time estimates are around or within the Quaternary period, a period that was marked by repeated growth and decay of ice sheets, causing alternate glacial and interglacial periods (Hewitt, 2004). It is well established that these glaciation cycles have affected the distribution of the species that we see in the Holarctic region (Hewitt, 2004; Hewitt, 2000). Perhaps the presence of ice sheets for extended durations, in combination with the large geographic distances between regions, reduced gene flow between North America and Europe, and have contributed to the genetic differentiation shown in these populations. Although migration, and even mass migration, has been documented for all the four taxa, this is believed to happen mostly within populations in restricted geographic regions rather than populations across continents (Dumont & Hinnekint, 1973; Wojtusiak, 1974; Michiels & Dhondt, 1991; Parr, 1997; Nikula, Sones & Trimble, 2001; Corbet, Longfeild & Moore, 1960). The deep divergence between North American and European populations here supports the idea that trans-Atlantic migrations are not common among dragonflies. This pattern of Palearctic and Nearctic divide has been observed in Butterflies as well (Maresova et al., 2019; Todisco et al., 2012), a group with comparable dispersal ability to dragonflies.

In addition, our results suggest that European populations have less genetic diversity compared to North America (Fig. 1, Table S4). In *L. quadrimaculata*, for example (Fig. 1D), of the total 30 haplotypes, we find that 17 haplotypes are North American while only five are European. Among these five haplotypes most are rare, with the majority of individuals sharing Haplotype #27. The remaining four haplotypes differ by only a single nucleotide. North American samples, on the other hand, exhibit many haplotypes that differ by often multiple nucleotides. In *A. juncea*, European samples have a comparable number of haplotypes to North America. However, as in *L. quadrimaculata*, most of these haplotypes differ from each other by only one nucleotide. Perhaps, particularly in the case of *A. juncea*, this lower genetic diversity in European populations might suggest a North American origin of these species, which then dispersed to Europe and became isolated there. Alternatively, or additionally, a lack of diversity in European populations could suggest severe effects on population sizes during glaciation cycles, leading to genetic drift losses of alleles (Galbreath & Cook, 2004). These taxa presumably existed at high latitudes; hence they would have been greatly impacted by the ice sheets which covered much of the northern Holarctic. Ice cover in the Beringia region was less than elsewhere in the Holarctic, however, which may have allowed for more refugia, further influencing differences in gene flow among the European and North American regions (Hewitt, 2004; Hewitt, 2000). Yet, another reason for lack of genetic variation in Europe might simply be an artefact of small sample size, which is insufficient to capture the diversity across the populations. Insects that have more widespread ranges in the Palearctic do show a large amount of genetic variation (Hewitt, 2004; Hewitt, 2000; Maresova et al., 2019; Simonsen, Olsen & Djernæs, 2020) which has been primarily attributed to mountain ranges in Europe. However, in such cases the species are usually considered to have southern ranges and post-glacial colonization takes places from these southern Refugia (Assmann, Nolte & Reuter, 1994; Hewitt, 2004; Hewitt, 1999; Sternberg, 1998; Simonsen, Olsen & Djernæs, 2020).

### Muddle in the middle: the “Greater Beringia” region

Another common pattern we find is the presence of an interesting geographic structure between populations from Japan, China and the Beringian region (far eastern Russia, Alaska and Yukon), which we collectively refer to as “Greater Beringia” region here. A mixed haplotype distribution is exhibited by populations across all species (excluding *S. sahlbergi*) in the Greater Beringia region. For example, take haplotype distribution of individuals from Japan. In *A. juncea* (Fig. 1A), Japanese individuals exhibit five different haplotypes: two of these haplotypes seem to be genetically different form both European and North American haplotypes, two are genetically close to North American haplotypes, and one haplotype is shared by samples from North America, Yukon–Alaska and Russia. Meanwhile, in *A. subarctica*, individuals from Japan only share haplotypes with Europe. In *L. quadrimaculata*, none of out the three Japanese haplotypes are shared by either North America or European individuals. Additionally, based on the clustering and divergence time estimation analysis, the Japanese samples for *A. juncea* and *S. danae* were shared within both North American and European populations (Figs. 2 and 3). On the contrary, for *A.subarctica* and *L. quadrimaculata* the Japanese samples seem to be more closely related to the European samples (Figs. 2 and 3). Similarly, across *A. subarctica, A. juncea, S. danae*, and *L. quadrimaculata* individuals from Russia, and Yukon–Alaska are either genetically close to North American samples, or to European samples, or are completely different from either depending on the species (Fig. 1). Lastly, for each species, we found that populations from China were usually genetically distinct from other populations.

While these geographic patterns have not been documented at the population level in insects before, these findings are by no means unprecedented. In a study of brown bear (*Ursus arctos*) populations in the Holarctic, Korsten et al. (2009) found that the population from Japan was actually divided in three different haplotypes. One of the haplotypes was found to be closely related to European populations, the second was found to group with Alaskan population, and the third haplotype was found to be completely different from all the other brown bear populations (Fig. 1b in Korsten et al. (2009)). A similar pattern in Japanese samples (four dragonfly species examined here) have also been observed in red fox (*Vulpes vulpes*) (Kutschera et al., 2013). This pattern in Brown bear and Red Fox are hypothesized to be the result of different waves of colonization to Japan and Beringian region in general.

To better understand the chaotic arrangement of haplotypes from the Greater Beringian region, one can treat this region as the middle of a geographic range with North America and Europe comprising its ends. Individuals from North America and Europe exhibit greater genetic differences between each other, as would be expected from individuals at each end of a species range (Eckert, Samis & Lougheed, 2008). However, individuals from the middle, i.e., the Greater Beringian region, perhaps never experience complete isolation from gene flow from either side of the larger range. Additionally, during the Quaternary, because of the cyclical glacial and interglacial periods, this region experienced fluctuating sea levels (Hewitt, 2000). As the sea levels decreased, land exposure increased, thereby increasing migratory opportunities and gene flow among dragonfly populations. By contrast, an increase in sea level would have reduced the amount of exposed land, thereby decreasing migration opportunities for dragonfly species, which generally are unlikely to cross sea water for long distances (with the exception of *Pantala* (Troast et al., 2016; Hobson et al., 2012), and *Hemianax* (Jensen & Nielsen, 2012; Norling, 1967)). Additionally, any increase and decrease of sea levels and temperature fluctuations during glaciation will also influence the availability of suitable habitat for these species. These species are known to inhabit in bogs and marshes in the boreal regions (Cannings & Cannings, 1985); habitats that are influenced by temperature and sea level fluctuations (Fronzek et al., 2010; Luoto, Heikkinen & Carter, 2004). Decrease in suitable habitat will increase insolation between populations leading to a decrease in geneflow. Perhaps this periodical increase and decrease in gene flow among populations explains the heterogeneity observed in haplotypes seen in the Greater Beringian region.

### Origins and biogeography

The origins of circumboreal taxa may be difficult to ascertain given the often complicated impacts of cyclical glaciation on populations and ranges. In addition to past glacial influence, the Holarctic has several potential barriers to dispersal, such as the Atlantic Ocean, the Bering Strait, and the Ural and Rocky Mountains, which may be insurmountable obstacles to some dragonflies and navigable to others. Over the geological history of the taxa studied here, at least part of the time the Bering Strait was instead a land bridge, Beringia. Clearly, given their circumboreal ranges, to the taxa studied here the Bering Strait and Beringia were not barriers to dispersal. From our data, we estimate using parsimony that *A. juncea* may have an East Asia origin, the region common among both major clades. It is possible that dispersal to the Nearctic and Palearctic occurred from East Asia, likely influenced by glaciation maxima and minima (this species was dated to be approximately 800 Kya, a time period with interglaciation, and much temperature fluctuations (PAGES, 2016)). The origin of *A. subarctica*, similar in divergence age to *A. juncea*, is more difficult to elucidate. Samples from Yukon and Alaska form the sister group of all other *A. juncea*. Nearctic specimens comprise the sister group of all the Palearctic specimens, suggesting a northwestern North America or Beringia origin. Our data suggests that the Beringian population became isolated before complete splitting of Palearctic and Nearctic populations occurred, possibly as a result of a dispersal event.

In *L. quadrimaculata*, a very old and divergent lineage (splitting ~1.5 Mya) from China forms the sister group of a clade comprising all other specimens. Remaining *L. quadrimaculata* samples form a clade comprised of two subclades, one exclusively Palearctic and one exclusively Nearctic. In the Palearctic subclade, the three Japanese specimens form the sister group of all the Western Palearctic specimens. As the earliest splits are lineages from China and Japan, this may suggest that *L. quadrimaculata* originated in East Asia and dispersed independently to the Nearctic and Western Palearctic regions. Low geographic coverage in East Asia (China and Japan) in our study makes it difficult to pinpoint its exact origin within East Asia. However, these results are concurrent with previous studies that have indeed suggested an Asian origin for *L. quadrimaculata* (Artiss, 2004).

*S. danae* is divided into Group A, which occurs only in the Nearctic region and Group B, which occurs in both the Nearctic and Palearctic regions. This may suggest that *S. danae* Group A+B originated and split in Western Nearctic, with Group A then spreading into the Eastern Nearctic, and Group B dispersing into the Palearctic.

### Odd one out: *Somatochlora sahlbergi*

The discussion so far has focused on the non-*Somatochlora* taxa in our study, which share similar patterns. Indeed, *S. sahlbergi* is the odd one out compared to the other four circumboreal dragonflies, *A. juncea*, *A. subarctica*, *L. quadrimaculata* and *S. danae* considered here. Unlike the other species, *S. sahlbergi* is genetically similar across its circumboreal range (Fig. 1). Kohli et al. (2018) hypothesized that one of the reasons for a lack of genetic diversity in *S. sahlbergi* might be a recent divergence age. Indeed, our results here show that *S. sahlbergi* diverged from its sister species *S. alpestris* around 1.8 Mya, i.e. in the Quaternary period. This age is younger than the divergence time estimates for three of the other species studied here; *A. juncea, A. subarctica* and *L. quadrimaculata* are older than *S. sahlbergi*, with an age of 2.6 My each. A recent divergence age would mean less time to accumulate mutations and hence lack of genetic differences in individuals across the continents. However, perhaps, it is not just the recent divergence time that alone contribute to the low genetic diversity in this species. Perhaps the answer also lies in the behavior of *S. sahlbergi*, particularly in terms of migration (or absence thereof) and niche plasticity.

There is evidence of recent migration for both *A. juncea* and *A. subarctica*. This is confirmed by one record of *A. juncea* (Wojtusiak, 1974) sampled in a location outside its present range although the ranges of these taxa are poorly sampled and defined. Similarly, *A. subarctica* was sampled 1995-1997 in a location in Massachusetts which is not part of its “typical” range as a boreal species found in peat bogs at higher latitudes, and it could not be found again 1998-1999 (Nikula, Sones & Trimble, 2001). There are several other species in the genus *Aeshna* that are known to migrate or disperse over long and short distances (Grewe et al., 2013; Schröter, 2011; Festi, 2011). Similarly, there is strong evidence of migration in *L. quadrimaculata* and *S. danae* as well (Parr, 1997; Michiels & Dhondt, 1991). However, there are no such migration records, long or short distance for *S. sahlbergi*. In fact, only very few short distance records exist for other members of genus *Somatochlora*. This might lead one to conclude that this species is a poor disperser. Further, migration of species and survival at a location outside their range indicate plasticity in habitat. For example, *A. subarctica* being found for three consecutive years in Massachusetts, a location that is not part of its current range. *L. quadrimaculata* on the other hand is known to have a large distribution, again suggesting that this species has a very broad niche. Having a broad niche is advantageous for surviving climatic fluctuations. There is evidence that suggests that *S. sahlbergi* might be a habitat generalist (Kohli et al., 2018) instead of a habitat specialist, however, if it is a poor disperser it will limit its ability to expand its range.

Lastly, there is very low divergence in *S. sahlbergi*, which might indicate a recent, rapid dispersal event following a genetic bottleneck (Kohli et al., 2018). As only *S. sahlbergi* specimens from the Yukon show any diversity, it is highly likely, based on the available material that the center of origin was northwestern North America or Beringia. This indicates that *S. sahlbergi* spread rapidly from a single, small glacial refugium following the last Glaciation. This potential pattern was also reported for the Boreal Eurasian damselfly *Nehalennia speciosa* (Carpentier) (Bernard et al., 2011).

### A new species of *Sympetrum danae*?

Given the amount of genetic diversity (6.67% genetically divergent) we see between Group A and Group B in *Sympetrum danae*, we believe that this might be evidence of a new species, as first suggested by Pilgrim & Von Dohlen (2012). Here, however, we show that among these cryptic taxa some Nearctic samples group with the European group; this suggests that rather than the two species simply being a European and Nearctic species, the actual species distribution patterns are more complex. It is beyond the scope of the present paper to describe the new species. We do not yet know of morphological variation between these two putative species, but the genetic separation seen here is known to similarly exist in the ITS marker (Pilgrim, unpublished data).

## Conclusions

First, we would like to acknowledge this study is limited in terms of taxon and gene sampling and therefore limited in its inference. We include as many available locations, as possible for each species, to closely represent the Holarctic region. This however means that for certain places, like Asia we have less taxon sampling (which is an artifact of obtaining most of the data from GenBank). Yet, our results shed light on the interesting patterns present in Asia and provide groundwork for a much larger study. Similarly, we also make a tradeoff between taxon sampling vs. multiple markers. Using only a single marker vastly increased the number of samples we can use in our analysis. The downside being that our results are from one marker and might change upon addition of more markers. Additionally, because of possible introgression in the mtDNA might obscure results at a phylogeographic level. However, our results don’t show any clear introgression. Despite this, our results provide groundwork for a much large comparative study using genomic random markers such as, RADseq. So, while we recognize these limitations, we argue that this study advances our understanding of circumboreal dragonflies and forms a great foundation for extensively exploring evolutionary patterns in Holarctic invertebrates.

Our results demonstrate that circumboreal and circumarctic insects may be very important for understanding phylogeographical patterns and processes linked to Quaternary glaciation cycles and climate change. Several Odonata in the suborder Zygoptera (damselflies) may display similar circumboreal distribution patterns to Anisoptera (dragonflies) species studied here and other insects like Lepidoptera. Such species should be the focus of future studies to obtain a fuller picture of circumboreal insect phylogeography, and especially the role of the Beringian region.

The deep divergence between North American and Beringian/Eurasian populations of *S. danae* indicate that the North American populations may represent a separate species. Assessing the status of these populations and describing a potential new species fall outside the scope of the current study but will be addressed in the future.

## Supplemental Information

10.7717/peerj.11338/supp-1 Supplemental Information 1 Supplementary information.

## Additional Information and Declarations

### Data Availability

The following information was supplied regarding data availability: Sequences are available at NCBI: MN847780 to MN847887.
