# Supplementary material for: Comparative phylogeography uncovers evolutionary past of Holarctic dragonflies
Source: PeerJ. 2021 Jun 24;9:e11338. doi: 10.7717/peerj.11338 (PMC8236228; doi:10.7717/peerj.11338)
Supplement: Supplemental Information 1 [file peerj-09-11338-s001.docx]

**Comparative phylogeography uncovers evolutionary past of circumboreal dragonflies**

Manpreet K. Kohli^*^, Marie Djernæs, Melissa Sanchez-Herrera, Göran Sahlén, Thomas Simonsen, Kent Olsen, Jessica L. Ware

*corresponding author

**Supplementary Information**

**Table of Contents**

S1 Table. List of specimens used in haplotype study 2
S2 Table. List of COI primers 11

S1 Appendix. Divergence time estimation methodology 12

S3.1 Table 13

S3.2 Table 18

S3.3 Table 19

S4 Table. Polymorphism statistics for the five species of Holarctic dragonflies 20

S1 Figure . Value of BIC versus number of clusters 22

S2 Figure . Density plot along Discriminant function 1 23

References 24

**Table S1. List of specimens used in haplotype study.**

| **Species/**  **Study ID** | **Source** | **GENBANK/**  **BOLD Accession**  **No. (CO1)** | **Voucher number** | **Collection Locality Country** | **Collection Locality State/Province** |
| --- | --- | --- | --- | --- | --- |
| ***Aeshna juncea*** | | | | | |
| JAP-1 | GenBank | AB708588 |  | Japan |  |
| JAP-2 | GenBank | AB708587 |  | Japan |  |
| JAP-3 | GenBank | AB708590 |  | Japan |  |
| RU-1 | GenBank | AB711463 |  | Russia |  |
| EU_FI-1 | GenBank | AB711458 |  | Finland |  |
| EU_NE-1 | GenBank | KF369278 |  | Netherlands |  |
| CA_Man-1 | GenBank | KR142479 |  | Canada |  |
| CA_Man-11 | GenBank | KR142266 |  | Canada |  |
| CA_Man-15 | GenBank | KR142917 |  | Canada |  |
| CA_Man-16 | GenBank | KR141108 |  | Canada |  |
| CA_Man-17 | GenBank | KR144661 |  | Canada |  |
| CA_Man-18 | GenBank | KR142375 |  | Canada |  |
| CA_Man-19 | GenBank | KR140582 |  | Canada |  |
| CA_Man-20 | GenBank | KR144938 |  | Canada |  |
| CA_Man-21 | GenBank | KR142385 |  | Canada |  |
| CA_Man-2 | GenBank | KR146790 |  | Canada |  |
| CA_Man-3 | GenBank | KR143341 |  | Canada |  |
| CA_Man-4 | GenBank | KR142506 |  | Canada |  |
| CA_Man-5 | GenBank | KR147910 |  | Canada |  |
| CA_Sask-1 | GenBank | JN294388 |  | Canada |  |
| CA_Man-6 | GenBank | KR142592 |  | Canada |  |
| CA_Man-7 | GenBank | KR147641 |  | Canada |  |
| CA_Man-8 | GenBank | KR143717 |  | Canada |  |
| CA_Man-9 | GenBank | KR144353 |  | Canada |  |
| CA_Man-10 | GenBank | KR148296 |  | Canada |  |
| CA_Man-12 | GenBank | KR148626 |  | Canada |  |
| CA_Man-13 | GenBank | KR144886 |  | Canada |  |
| CA_Man-14 | GenBank | KR145533 |  | Canada |  |
| CA_Sask-2 | GenBank | JN294385 |  | Canada |  |
| CA_Sask-3 | GenBank | JN294387 |  | Canada |  |
| YukT-1 | BOLD systems | ODRMA036-10 |  | Canada |  |
| YukT-2 | BOLD systems | ODRMA037-10 |  | Canada |  |
| YukT-3 | BOLD systems | ODRMA038-10 |  | Canada |  |
| CA_BritC-1 | BOLD systems | ODRMA083-10 |  | Canada |  |
| RU_Sakls-1 | GenBank | AB708586 |  | Russia |  |
| JAP-4 | GenBank | AB708581 |  | Japan |  |
| RU-3 | GenBank | AB708591 |  | Russia |  |
| RU-4 | GenBank | AB708582 |  | Russia |  |
| RU-5 | GenBank | AB708583 |  | Russia |  |
| JAP-5 | GenBank | AB708589 |  | Japan |  |
| JAP-6 | GenBank | AB708584 |  | Japan |  |
| JAP_SK-1 | GenBank | AB711464 |  | South Korea |  |
| EU_GER-1 | BOLD systems | FBAQU1431-13 |  | Germany |  |
| JAP-7 | GenBank | AB708585 |  | Japan |  |
| EU_GER-2 | BOLD systems | FBAQU479-10 |  | Germany |  |
| Alas-1 | BOLD systems | UAMIC785-13 |  | USA | Alaska |
| Alas-2 | BOLD systems | UAMIC837-13 |  | USA | Alaska |
| EU_DE-8 | This study | MN847866 | ENT-DNA-131 | Denmark | Gribskov, Skallerød Vang |
| EU_DE-9 | This study | MN847867 | ENT-DNA-189 | Denmark | Letmose, large pond |
| EU_DE-10 | This study | MN847868 | ENT-DNA-249 | Denmark | Wistoft Plantage, Karup |
| EU_DE-11 | This study | MN847869 | ENT-DNA-250 | Denmark | Ulvedal, Karup |
| EU_DE-12 | This study | MN847870 | ENT-DNA-251 | Denmark | Rømø Kirkeby |
| EU_DE-13 | This study | MN847871 | ENT-DNA-252 | Denmark | Lille Vildmose, Portlandsmosen |
| EU_DE-14 | This study | MN847872 | ENT-DNA-253 | Denmark | Lille Vildmose, Portlandsmosen |
| EU_DE-15 | This study | MN847873 | ENT-DNA-668 | Denmark | Vejers |
| EU_SW-1 | This study | MN847874 | ENT-DNA-955 | Sweden | So. Sodertalje Kn. |
| EU_FR-1 | This study | MN847875 | ENT-DNA-973 | France | Sources du Tarn River, L Hopital, le Pont de Montvert |
| EU_FR-2 | This study | MN847876 | ENT-DNA-974 | France | Sources du Tarn River, L Hopital, le Pont de Montvert |
| EU_AU-1 | This study | MN847877 | ENT-DNA-976 | Austria | Sinesbrunn Moore, Tyrol |
| EU_FR-3 | This study | MN847878 | ENT-DNA-998 | France | la Plain du Tarn River, L Hopital, le Pont de Montvert |
| EU_DE-1 | This study | MN847879 | ENT-DNA-1040 | Denmark | Lille Vildmose, Portlandsmosen |
| EU_DE-2 | This study | MN847880 | ENT-DNA-1041 | Denmark | Letmose, large pond |
| EU_DE-3 | This study | MN847881 | ENT-DNA-1042 | Denmark | Letmose, large pond |
| EU_DE-4 | This study | MN847882 | ENT-DNA-1043 | Denmark | Sepstrup Sande |
| EU_DE-5 | This study | MN847883 | ENT-DNA-1157 | Denmark | Badesøen, Munkekær, Munkekaer, Læsø Klitplantage |
| EU_DE-6 | This study | MN847884 | ENT-DNA-1158 | Denmark | Badesøen, Munkekær, Munkekaer, Læsø Klitplantage |
| EU_DE-7 | This study | MN847885 | ENT-DNA-1159 | Denmark | Store Hjøllund Plantage |
| EU_FI-2 | This study | MN847886 | ENT-DNA-1160 | Finland | Pikku Sorlampi |
| EU_FI-3 | This study | MN847887 | ENT-DNA-1161 | Finland | Pikku Sorlampi |
| CH-1 | GenBank | MF358804.1 |  | China |  |
| CH-2 | GenBank | MF358805.1 |  | China |  |
| EU_GER-4 | GenBank | KU180297 |  | Germany |  |
| CA_NorthT-1 | This study | MN847832 | EMO1 | Canada | Northwest Territory |
| CA_NorthT-2 | This study | MN847833 | EMO2 | Canada | Northwest Territory |
| YukT-12 | This study | MN847834 | EMO5 | Canada | Yukon Territory |
| YukT-13 | This study | MN847835 | EMO6 | Canada | Yukon Territory |
| YukT-14 | This study | MN847836 | EMO9 | Canada | Yukon Territory |
| YukT-4 | This study | MN847837 | EMO14 | Canada | Yukon Territory |
| YukT-5 | This study | MN847838 | EMO15 | Canada | Yukon Territory |
| YukT-6 | This study | MN847839 | EMO17 | Canada | Yukon Territory |
| YukT-7 | This study | MN847840 | EMO18 | Canada | Yukon Territory |
| YukT-8 | This study | MN847841 | EMO22 | Canada | Yukon Territory |
| YukT-9 | This study | MN847842 | EMO23 | Canada | Yukon Territory |
| YukT-10 | This study | MN847843 | EMO26 | Canada | Yukon Territory |
| YukT-11 | This study | MN847844 | EMO32 | Canada | Yukon Territory |
| CA_NorthT-3 | This study | MN847845 | EMO34 | Canada | Northwest Territory |
| CA_Quebec-1 | This study | MN847846 | EMO41 | Canada | Quebec |
| CA_Quebec-2 | This study | MN847847 | EMO42 | Canada | Branchville |
| EU_SW-2 | This study | MN847848 | JLWS1 | Sweden | Sweden |
| EU_SW-3 | This study | MN847849 | JLWS4 | Sweden | Sweden |
| EU_SW-4 | This study | MN847850 | JLWS5 | Sweden | Sweden |
| RU-6 | This study | MN847851 | JLWS6 | Russia | Russia |
| EU_NOR-1 | This study | MN847852 | JLWS8 | Norway | Norway |
| CA_BritC-2 | This study | MN847853 | LR29 | Canada | British Columbia |
| CA_BritC-3 | This study | MN847854 | LR30 | Canada | British Columbia |
| RU-13 | This study | MN847855 | KM6 | Russia | Piy-Khemskiy Rayon, Tuva |
| RU-14 | This study | MN847856 | KM7 | Russia | Piy-Khemskiy Rayon, Tuva |
| RU-15 | This study | MN847857 | KM8 | Russia | Piy-Khemskiy Rayon, Tuva |
| RU-16 | This study | MN847858 | KM9 | Russia | Piy-Khemskiy Rayon, Tuva |
| RU-7 | This study | MN847859 | KM10 | Russia | Piy-Khemskiy Rayon, Tuva |
| RU-8 | This study | MN847860 | KM11 | Russia | Piy-Khemskiy Rayon, Tuva |
| RU-9 | This study | MN847861 | KM12 | Russia | Piy-Khemskiy Rayon, Tuva |
| RU-10 | This study | MN847862 | KM13 | Russia | Piy-Khemskiy Rayon, Tuva |
| RU-11 | This study | MN847863 | KM14 | Russia | Piy-Khemskiy Rayon, Tuva |
| RU-12 | This study | MN847864 | KM18 | Russia | Piy-Khemskiy Rayon, Tuva |
| CA_Utah-1 | This study | MN847865 | KM51 | USA | Summit Co. Utah |
| ***Aeshna subarctica*** | | | | | |
| Sask-1 | GenBank | JN294386 |  | Canada | Saskatchewan |
| Man-1 | GenBank | KR143264 |  | Canada | Manitoba |
| Man-2 | GenBank | KR147009 |  | Canada | Manitoba |
| JAP-1 | GenBank | AB711461 |  | Japan | Hokkaido,Bekkai |
| JAP-2 | GenBank | AB711459 |  | Japan | Hokkaido,Kamikawa |
| JAP-3 | GenBank | AB708595 |  | Japan | Hokkaido,Bekkai |
| FI-1 | GenBank | AB711462 |  | Finland |  |
| JAP-4 | GenBank | AB711460 |  | Japan | Hokkaido |
| GER-1 | GenBank | KU180298 |  | Germany |  |
| YukT-1 | GenBank | JF839258 |  | Canada | Yukon Territory |
| YukT-2 | GenBank | JF839259 |  | Canada | Yukon Territory |
| YukT-3 | GenBank | JF839332 |  | Canada | Yukon Territory |
| BritC-1 | GenBank | JF839363 |  | Canada | British Columbia |
| Alas-1 | GenBank | KU873995 |  | USA | Alaska |
| Alas-2 | GenBank | KU873996 |  | USA | Alaska |
| RUS-1 | This Study | MN847780 | KM43 | Russia | Novosibirsk |
| GER-2 | GenBank | FBAQU521-10 |  | Germany | Bavaria |
| DEN-1 | This Study | MN847781 | ENT-DNA-132 | Denmark | Gribskov, Hovmosen |
| DEN-2 | This Study | MN847782 | ENT-DNA-133 | Denmark | Tokkerup Tørvemose |
| DEN-3 | This Study | MN847783 | ENT-DNA-134 | Denmark | Gribskov, Sandskredsø |
| DEN-4 | This Study | MN847784 | ENT-DNA-187 | Denmark | Letmose, lille vandhul |
| DEN-5 | This Study | MN847785 | ENT-DNA-188 | Denmark | Letmose, lille vandhul |
| DEN-6 | This Study | MN847786 | ENT-DNA-190 | Denmark | Letmose, lille vandhul |
| DEN-7 | This Study | MN847787 | ENT-DNA-653 | Denmark | Tversted Rimmer |
| DEN-8 | This Study | MN847788 | ENT-DNA-654 | Denmark | Tversted Rimmer |
| DEN-9 | This Study | MN847789 | ENT-DNA-655 | Denmark | Hovmosen, Gadevang |
| DEN-10 | This Study | MN847790 | ENT-DNA-656 | Denmark | Hovmosen, Gadevang |
| DEN-11 | This Study | MN847791 | ENT-DNA-657 | Denmark | Hovmosen, Gadevang |
| SW-1 | This Study | MN847792 | ENT-DNA-956 | Sweden | Södertälje Kn. Lillsjön |
| SW-2 | This Study | MN847793 | ENT-DNA-957 | Sweden | Södertälje Kn. Lillsjön |
| NOR-1 | This Study | MN847794 | ENT-DNA-962 | Norway | Hvaler, NE Sandberg |
| GER-3 | This Study | MN847795 | ENT-DNA-972 | Germany | Dosenmoor |
| FI-2 | This Study | MN847796 | ENT-DNA-1163 | Finland | Pikku Sorlampi |
| ***Libellula quadrimaculata*** | | | | | |
| NewFL-1 | GenBank | HM413521 |  | Canada | Newfoundland and Labrador |
| NewFL-2 | GenBank | HM413529 |  | Canada | Newfoundland and Labrador |
| NewFL-3 | GenBank | HM413566 |  | Canada | Newfoundland and Labrador |
| BritC-1 | GenBank | MG378286 |  | Canada | British Columbia |
| Ont-1 | GenBank | MG376152 |  | Canada | Ontario |
| Alberta-1 | GenBank | JN294338 |  | Canada | Alberta |
| SasK-1 | GenBank | JN294400 |  | Canada | Saskatchewan |
| SasK-2 | GenBank | JN294401 |  | Canada | Saskatchewan |
| SasK-3 | GenBank | JN294403 |  | Canada | Saskatchewan |
| SasK-4 | GenBank | JN294404 |  | Canada | Saskatchewan |
| SasK-5 | GenBank | JN294437 |  | Canada | Saskatchewan |
| SasK-6 | GenBank | JN294439 |  | Canada | Saskatchewan |
| SasK-7 | GenBank | JN294440 |  | Canada | Saskatchewan |
| SasK-8 | GenBank | JN294441 |  | Canada | Saskatchewan |
| Alberta-2 | GenBank | MG463189 |  | Canada | Alberta |
| Alberta-3 | GenBank | KR919033 |  | Canada | Alberta |
| Alberta-4 | GenBank | KR919420 |  | Canada | Alberta |
| Ont-2 | GenBank | KR144312 |  | Canada | Ontario |
| Ont-3 | GenBank | HM399588 |  | Canada | Ontario |
| Ont-4 | GenBank | HM399589 |  | Canada | Ontario |
| Ont-5 | GenBank | HM399590 |  | Canada | Ontario |
| NewB-1 | GenBank | JN419954 |  | Canada | New Brunswick |
| GER-1 | GenBank | HM901870 |  | Germany | Bavaria |
| Unknown-2 | GenBank | KF257060 |  |  |  |
| JAP-1 | GenBank | AB708986 |  | Japan |  |
| JAP-2 | GenBank | AB708987 |  | Japan | Hokkaido |
| JAP-3 | GenBank | AB708985 |  | Japan |  |
| YukT-1 | GenBank | JF839248 |  | Canada | Yukon Territory |
| YukT-2 | GenBank | JF839249 |  | Canada | Yukon Territory |
| BritC-2 | GenBank | JF839307 |  | Canada | British Columbia |
| BritC-3 | GenBank | JF839308 |  | Canada | British Columbia |
| Ont-6 | GenBank | KM533579 |  | Canada | Ontario |
| Ont-7 | GenBank | KM531593 |  | Canada | Ontario |
| Ont-8 | GenBank | KM537510 |  | Canada | Ontario |
| Ont-9 | GenBank | KM528898 |  | Canada | Ontario |
| Ont-10 | GenBank | KM536673 |  | Canada | Ontario |
| Ont-11 | GenBank | KM531766 |  | Canada | Ontario |
| Ont-12 | GenBank | KM530724 |  | Canada | Ontario |
| Ont-13 | GenBank | MG381893 |  | Canada | Ontario |
| Ont-14 | BOLD system | TZBCA545-07 |  | Canada | Ontario |
| Ont-15 | BOLD system | TZBCA546-07 |  | Canada | Ontario |
| Alas-1 | GenBank | KU875374 |  | USA | Alaska |
| Alas-2 | GenBank | KU875373 |  | USA | Alaska |
| DEN-1 | This Study | MN847818 | ENT-DNA-401 | Denmark | Bastemosen |
| DEN-2 | This Study | MN847819 | ENT-DNA-426 | Denmark | Gribskov, Skallerød Vang |
| DEN-3 | This Study | MN847820 | ENT-DNA-429 | Denmark | Vråby Plantage, Rømø |
| DEN-4 | This Study | MN847821 | ENT-DNA-430 | Denmark | Vråby Plantage, Rømø |
| DEN-5 | This Study | MN847822 | ENT-DNA-431 | Denmark | Vejers |
| DEN-6 | This Study | MN847823 | ENT-DNA-432 | Denmark | Vejers |
| DEN-7 | This Study | MN847824 | ENT-DNA-433 | Denmark | Tørvehul, Borris Sønderland |
| DEN-8 | This Study | MN847825 | ENT-DNA-434 | Denmark | Krapdiget, Vejlerne |
| DEN-9 | This Study | MN847826 | ENT-DNA-435 | Denmark | Ejby Mose |
| DEN-10 | This Study | MN847827 | ENT-DNA-436 | Denmark | Ejby Mose |
| DEN-11 | This Study | MN847828 | ENT-DNA-437 | Denmark | Store Hareskov |
| DEN-12 | This Study | MN847829 | ENT-DNA-438 | Denmark | Borremose |
| DEN-13 | This Study | MN847830 | ENT-DNA-439 | Denmark | Safirsøen |
| DEN-14 | This Study | MN847831 | ENT-DNA-440 | Denmark | Sortemyr |
| GER-2 | GenBank | LC366713 |  | Germany |  |
| CH-1 | GenBank | MF358747 |  | China | Ningbo |
| CH-2 | GenBank | MF358748 |  | China | Ningbo |
| ***Sympetrum danae*** | | | | | |
| RUS-2 | GenBank | EU243890 |  | Russia |  |
| YukT-11 | GenBank | EU243914 |  | Canada | Yukon Territory |
| YukT-10 | GenBank | EU243913 |  | Canada | Yukon Territory |
| YukT-3 | GenBank | EU243912 |  | Canada | Yukon Territory |
| YukT-2 | GenBank | EU243911 |  | Canada | Yukon Territory |
| Wis-10 | GenBank | EU243910 |  | USA | Wisconsin |
| Was-06 | GenBank | EU243909 |  | USA | Washington |
| Was-05 | GenBank | EU243908 |  | USA | Washington |
| Was-04 | GenBank | EU243907 |  | USA | Washington |
| Was-03 | GenBank | EU243906 |  | USA | Washington |
| Utah-15 | GenBank | EU243905 |  | USA | Utah |
| Utah-14 | GenBank | EU243904 |  | USA | Utah |
| Utah-12 | GenBank | EU243903 |  | USA | Utah |
| Utah-11 | GenBank | EU243902 |  | USA | Utah |
| Utah-10 | GenBank | EU243901 |  | USA | Utah |
| Utah-09 | GenBank | EU243900 |  | USA | Utah |
| Utah-08 | GenBank | EU243899 |  | USA | Utah |
| Utah-07 | GenBank | EU243898 |  | USA | Utah |
| Utah-06 | GenBank | EU243897 |  | USA | Utah |
| Utah-05 | GenBank | EU243896 |  | USA | Utah |
| Utah-04 | GenBank | EU243895 |  | USA | Utah |
| Utah-03 | GenBank | EU243894 |  | USA | Utah |
| Utah-02 | GenBank | EU243893 |  | USA | Utah |
| Utah-01 | GenBank | EU243892 |  | USA | Utah |
| Oreg-05 | GenBank | EU243889 |  | USA | Oregon |
| Oreg-04 | GenBank | EU243888 |  | USA | Oregon |
| Oreg-03 | GenBank | EU243887 |  | USA | Oregon |
| Oreg-02 | GenBank | EU243886 |  | USA | Oregon |
| Oreg-01 | GenBank | EU243885 |  | USA | Oregon |
| NorthT-2 | GenBank | EU243884 |  | Canada | Northwest Territories |
| NorthT-1 | GenBank | EU243883 |  | Canada | Northwest Territories |
| Mich-40 | GenBank | EU243882 |  | USA | Michigan |
| Mich-39 | GenBank | EU243881 |  | USA | Michigan |
| Mich-13 | GenBank | EU243880 |  | USA | Michigan |
| Mich-12 | GenBank | EU243879 |  | USA | Michigan |
| Mich-11 | GenBank | EU243878 |  | USA | Michigan |
| Mich-10 | GenBank | EU243877 |  | USA | Michigan |
| Mich-09 | GenBank | EU243876 |  | USA | Michigan |
| Mich-08 | GenBank | EU243875 |  | USA | Michigan |
| Mich-07 | GenBank | EU243874 |  | USA | Michigan |
| Mich-06 | GenBank | EU243873 |  | USA | Michigan |
| BritC-17 | GenBank | EU243872 |  | Canada | British Columbia |
| BritC-16 | GenBank | EU243871 |  | Canada | British Columbia |
| BritC-15 | GenBank | EU243870 |  | Canada | British Columbia |
| BritC-14 | GenBank | EU243869 |  | Canada | British Columbia |
| BritC-13 | GenBank | EU243868 |  | Canada | British Columbia |
| BritC-12 | GenBank | EU243867 |  | Canada | British Columbia |
| BritC-11 | GenBank | EU243866 |  | Canada | British Columbia |
| BritC-10 | GenBank | EU243865 |  | Canada | British Columbia |
| BritC-8 | GenBank | EU243864 |  | Canada | British Columbia |
| BritC-7 | GenBank | EU243863 |  | Canada | British Columbia |
| BritC-6 | GenBank | EU243862 |  | Canada | British Columbia |
| BritC-5 | GenBank | EU243861 |  | Canada | British Columbia |
| BritC-4 | GenBank | EU243860 |  | Canada | British Columbia |
| BritC-3 | GenBank | EU243859 |  | Canada | British Columbia |
| BritC-2 | GenBank | EU243858 |  | Canada | British Columbia |
| BritC-1 | GenBank | EU243857 |  | Canada | British Columbia |
| Alas-02 | GenBank | EU243856 |  | USA | Alaska |
| Alas-01 | GenBank | EU243855 |  | USA | Alaska |
| YukT-12 | GenBank | EU243854 |  | Canada | Yukon Territory |
| YukT-9 | GenBank | EU243853 |  | Canada | Yukon Territory |
| YukT-8 | GenBank | EU243852 |  | Canada | Yukon Territory |
| YukT-7 | GenBank | EU243851 |  | Canada | Yukon Territory |
| YukT-6 | GenBank | EU243850 |  | Canada | Yukon Territory |
| YukT-5 | GenBank | EU243849 |  | Canada | Yukon Territory |
| YukT-4 | GenBank | EU243848 |  | Canada | Yukon Territory |
| YukT-1 | GenBank | EU243847 |  | Canada | Yukon Territory |
| SakIs-1 | GenBank | EU243846 |  | Russia | Sakhalin |
| Rus-8 | GenBank | EU243845 |  | Russia |  |
| Rus-7 | GenBank | EU243844 |  | Russia |  |
| Rus-6 | GenBank | EU243843 |  | Russia |  |
| Rus-5 | GenBank | EU243842 |  | Russia |  |
| Rus-4 | GenBank | EU243841 |  | Russia |  |
| Rus-1 | GenBank | EU243840 |  | Russia |  |
| Neth-43 | GenBank | EU243839 |  | Netherlands |  |
| Neth-42 | GenBank | EU243838 |  | Netherlands |  |
| Neth-41 | GenBank | EU243837 |  | Netherlands |  |
| Ire-36 | GenBank | EU243836 |  | Ireland |  |
| Ire-35 | GenBank | EU243835 |  | Ireland |  |
| Ire-34 | GenBank | EU243834 |  | Ireland |  |
| Ire-33 | GenBank | EU243833 |  | Ireland |  |
| Ire-32 | GenBank | EU243832 |  | Ireland |  |
| Ire-31 | GenBank | EU243831 |  | Ireland |  |
| Bela-38 | GenBank | EU243830 |  | Belarus |  |
| Bela-37 | GenBank | EU243829 |  | Belarus |  |
| BritC-18 | GenBank | EU243828 |  | Canada | British Columbia |
| BritC-9 | GenBank | EU243827 |  | Canada | British Columbia |
| Alas-30 | GenBank | EU243826 |  | USA | Alaska |
| Alas-27 | GenBank | EU243825 |  | USA | Alaska |
| Alas-26 | GenBank | EU243824 |  | USA | Alaska |
| Alas-25 | GenBank | EU243823 |  | USA | Alaska |
| Alas-17 | GenBank | EU243822 |  | USA | Alaska |
| Alas-16 | GenBank | EU243821 |  | USA | Alaska |
| Alas-15 | GenBank | EU243820 |  | USA | Alaska |
| Alas-14 | GenBank | EU243819 |  | USA | Alaska |
| Alas-13 | GenBank | EU243818 |  | USA | Alaska |
| Alas-12 | GenBank | EU243817 |  | USA | Alaska |
| Alas-11 | GenBank | EU243816 |  | USA | Alaska |
| RUS-12 | GenBank | EU743616 |  | Russia |  |
| RUS-10 | GenBank | EU743614 |  | Russia |  |
| RUS-11 | GenBank | EU743615 |  | Russia |  |
| RUS-9 | GenBank | EU743613 |  | Russia |  |
| Albrt-1 | GenBank | JN294355 |  | Canada | Alberta |
| Albrt-2 | GenBank | JN294359 |  | Canada | Alberta |
| Albrt-3 | GenBank | JN294378 |  | Canada | Alberta |
| SasK-1 | GenBank | JN294409 |  | Canada | Saskatchewan |
| SasK-2 | GenBank | JN294412 |  | Canada | Saskatchewan |
| SasK-3 | GenBank | JN294415 |  | Canada | Saskatchewan |
| SasK-4 | GenBank | JN294417 |  | Canada | Saskatchewan |
| SasK-5 | GenBank | JN294418 |  | Canada | Saskatchewan |
| SasK-6 | GenBank | JN294419 |  | Canada | Saskatchewan |
| SasK-7 | GenBank | JN294420 |  | Canada | Saskatchewan |
| SasK-8 | GenBank | JN294422 |  | Canada | Saskatchewan |
| SasK-9 | GenBank | JN294425 |  | Canada | Saskatchewan |
| SasK-10 | GenBank | JN294426 |  | Canada | Saskatchewan |
| SasK-11 | GenBank | JN294429 |  | Canada | Saskatchewan |
| SasK-12 | GenBank | JN294430 |  | Canada | Saskatchewan |
| SasK-13 | GenBank | JN294475 |  | Canada | Saskatchewan |
| BritC-19 | GenBank | JN294508 |  | Canada | British Columbia |
| BritC-20 | GenBank | JN294513 |  | Canada | British Columbia |
| GER-1 | GenBank | HM901878 |  | Germany | Bavaria |
| JAP-1 | GenBank | AB709122 |  | Japan |  |
| JAP-2 | GenBank | AB709120 |  | Japan | Hokkaido |
| JAP-3 | GenBank | AB709121 |  | Japan |  |
| BritC-21 | GenBank | JF839312 |  | Canada | British Columbia |
| YukT-13 | GenBank | JF839317 |  | Canada | Yukon Territory |
| YukT-14 | GenBank | JF839318 |  | Canada | Yukon Territory |
| Man-1 | GenBank | KM532431 |  | Canada | Manitoba |
| Man-2 | GenBank | KM531697 |  | Canada | Manitoba |
| Man-3 | GenBank | KM537729 |  | Canada | Manitoba |
| Man-4 | GenBank | KM534364 |  | Canada | Manitoba |
| Man-5 | GenBank | KM528837 |  | Canada | Manitoba |
| Ont-1 | GenBank | KM533093 |  | Canada | Ontario |
| Ont-2 | GenBank | KM535920 |  | Canada | Ontario |
| Ont-3 | GenBank | KM532019 |  | Canada | Ontario |
| Ont-4 | GenBank | KM528869 |  | Canada | Ontario |
| Ont-5 | GenBank | KM529124 |  | Canada | Ontario |
| Sask-14 | GenBank | KM532923 |  | Canada | Saskatchewan |
| DEN-1 | This Study | MN847797 | ENT-DNA-570.2 | Denmark | Kragemosen, Samsø |
| DEN-2 | This Study | MN847798 | ENT-DNA-572 | Denmark | Rønnerne, Læsø |
| DEN-3 | This Study | MN847799 | ENT-DNA-586 | Denmark | Vejers |
| DEN-4 | This Study | MN847800 | ENT-DNA-587 | Denmark | Røde Sø |
| DEN-5 | This Study | MN847801 | ENT-DNA-588 | Denmark | Lake north ofRåbjerg Mile |
| DEN-6 | This Study | MN847802 | ENT-DNA-589 | Denmark | Borris Sønderland |
| DEN-7 | This Study | MN847803 | ENT-DNA-590 | Denmark | Letmosen |
| DEN-8 | This Study | MN847804 | ENT-DNA-591 | Denmark | Wistoft Plantage, Karup |
| DEN-9 | This Study | MN847805 | ENT-DNA-592 | Denmark | Vråby Plantage, Rømø |
| DEN-10 | This Study | MN847806 | ENT-DNA-595 | Denmark | Kattehale Mose |
| DEN-11 | This Study | MN847807 | ENT-DNA-596 | Denmark | Gribskov v. station |
| DEN-12 | This Study | MN847808 | ENT-DNA-597 | Denmark | Holmegaards Mose, Tyvkrogen |
| DEN-13 | This Study | MN847809 | ENT-DNA-598 | Denmark | Holmegaards Mose, Tyvkrogen |
| DEN-14 | This Study | MN847810 | ENT-DNA-599 | Denmark | Bog south of Store Gribsø |
| FR-1 | This Study | MN847811 | ENT-DNA-600 | France | Kattehale Mose |
| FR-2 | This Study | MN847812 | ENT-DNA-1093 | France | Ètang de pêche de Barrandon, Les Laubies, Mende |
| GER-2 | This Study | MN847813 | ENT-DNA-1094 | Germany | Ètang de pêche de Barrandon, Les Laubies, Mende |
| GER-3 | This Study | MN847814 | ENT-DNA-1136 | Germany | Dosenmoor |
| DEN-15 | This Study | MN847815 | ENT-DNA-1137 | Germany | Dosenmoor |
| DEN-16 | This Study | MN847816 | ENT-DNA-1165 | Denmark | Badesøen, Munkekær, Læsø Klitplantage |
| DEN-17 | This Study | MN847817 | ENT-DNA-1166 | Denmark | Badesøen, Munkekær, Læsø Klitplantage |

**Table S2. List of COI primers.**

| Gene | Up Sequence | Up Source | Down Sequence | Down Source | Lab |
| --- | --- | --- | --- | --- | --- |
| COI | 5′ TAATTGGAGGA TTTGGAAATTG 3′ | Coi1709F, Wells and Sperling 1999 | 5′ CCYGGTARAATTA RAATRTARACTTC 3′ | COI2191R, Simon et al. 1994 | Newark, USA |
| COI | 5′ TTTCTACAAAYCAY  AARGATATTGG 3′ | OdoF2, Simonsen et al. in review | 5′ TAAACYTCTGGRT  GRCCAAARAATCA 3′ | OdoR3, Simonsen et al. in review | Aarhus, Denmark |

OdoF2 and OdoR3 were used as tailed primers, with universal primers (M13-FP and M13R-Puc) used as tails. The universal tails were in turn used as sequencing primers.

OdoF2_t1 = M13-FP + OdoF2:

5’ TGTAAAACGACGGCCAGTTTTCTACAAAYCAYAARGATATTGG 3’

OdoR3_t1 = M13R-Puc + OdoR3:

5’ CAGGAAACAGCTATGACTAAACYTCTGGRTGRCCAAARAATCA 3’

M13-FP:

5’ TGTAAAACGACGGCCAGT 3’

M13R-Puc:

5’ CAGGAAACAGCTATGAC 3’

**Appendix 1: Divergence time estimation methodology**

Besides looking at the genetic variation in the five species across the continents we also wanted to estimate divergence time between the populations. However, since no fossils are available for *Aeshna juncea*, *Aeshna subarctica*, *Somatochlora sahlbergi*, *Libellula quadrimaculata* or *Sympetrum danae* we can’t directly estimate the time of divergence between populations. We will have to use secondary calibration methods. To do that we first need to estimate divergence times within each of the genera *Aeshna*, *Somatochlora*, *Libellula* and *Sympetrum*. To accomplish that we performed the following steps for each genus:

1. Created alignments by sampling all species within each genus for which the COI gene sequence was recorded on either GenBank or BOLD system. Samples used in this step are listed in supplementary table S3.1
2. Partitioned the alignment based on codon position and then estimated the model of evolution for each of the partition using the software ModelFinder (Kalyaanamoorthy et al 2017) as implemented in IQ-Tree (Trifinopoulos et al 2016). Chose the most likely model for each of the partitions based on Bayesian Information Criterion (BIC), models are listed in supplementary table S3.2
3. Determined the oldest crown fossil for each genus, which was then used for establishing a time prior. Fossils used for each genera are listed in supplementary table S3.3
4. Performed divergence time estimation analysis in BEAST V2.5.2 (Suchard et al 2018). With the exception of *Libellula*, we estimated divergence times for each genus in *BEAST using a Coalescent Bayesian Skyline tree model and uncorrelated relaxed clock model. Divergence for *Libellula* was estimated in Standard BEAST under the Coalescent Bayesian Skyline tree prior, since we did not have enough sampling for each of the species in this genus. All the logs were tested to in TRACER 1.7 (Rambaut et al. 2018) for convergence after a burnin of 10%.

Based on the results of divergence time analyses described above, we established the age of the five species of interest. For example, from the chronogram recovered for genus *Aeshna*, we established the age of *Aeshna juncea* and *Aeshna subarctica*. These dates were then used as priors at the root for divergence time estimation analysis for each of the species. As above, we ran ModelFinder using BIC for sequence alignments for each of the species (supplementary table S3.2 for the best model for each species). Divergence time estimation analysis was conducted in Standard BEAST under the Coalescent Bayesian Skyline tree prior and uncorrelated relaxed clock model.

**Table S3.1.** **List of specimens used in divergence time estimation analysis for genera *Aeshna*, *Somatochlora* and *Libellula***

| **Alignment code** | | **Accession number /**  **Voucher number** |
| --- | --- | --- |
| **Genus *Aeshna*** | |  |
| ***Aeshna affinis*** | |  |
| affinis-1 | | KM25 |
| affinis-2 | | KM26 |
| affinis-3 | | KM29 |
| ***Aeshna canadensis*** | |  |
| canadensis-1 | | JN294474 |
| canadensis-2 | | HM413508 |
| canadensis-3 | | HM413507 |
| ***Aehsna constricta*** | |  |
| constricta-1 | | GU013562 |
| constricta-2 | | KM534106 |
| constricta-3 | | KM533275 |
| ***Aeshna crenata*** | |  |
| crenata-1 | | AB711457 |
| crenata-2 | | AB711456 |
| crenata-3 | | AB711455 |
| ***Aeshna cyanea*** | |  |
| cyanea-1 | | KU180321 |
| cyanea-2 | | KU180320 |
| cyanea-3 | | KU180319 |
| ***Aeshna eremita*** | |  |
| eremita-1 | | HM381225 |
| eremita-2 | | HM381222 |
| eremita-3 | | JN294526 |
| ***Aeshna grandis*** | |  |
| grandis-1 | | KJ873213 |
| grandis-2 | | KU180299 |
| grandis-3 | | KC912203 |
| ***Aeshna interrupta*** | |  |
| interrupta-1 | | HM381232 |
| interrupta-2 | | JN294525 |
| interrupta-3 | | JN294504 |
| ***Aeshna juncea*** | |  |
| juncea-1 | | JN294388 |
| juncea-2 | | JN294387 |
| juncea-3 | | JN294385 |
| ***Aeshna mixta*** | |  |
| mixta-1 | | KC912205 |
| mixta-2 | | KC912204 |
| mixta-3 | | AB708592 |
| ***Aeshna palmata*** | |  |
| palmata-1 | | JN294524 |
| palmata-2 | | JN294523 |
| palmata-3 | | JN294498 |
| ***Aeshna septentrionalis*** | |  |
| septentrionalis-1 | | GU714053 |
| septentrionalis-2 | | GU714052 |
| septentrionalis-3 | | KR143189 |
| ***Aeshna serrata*** | |  |
| serrata-1 | | AB708594 |
| serrata-2 | | AB708593 |
| ***Aeshna sitchensis*** | |  |
| sitchensis-1 | | HM413523 |
| sitchensis-2 | | KR142486 |
| sitchensis-3 | | KU873993 |
| ***Aeshna subarctica*** | |  |
| subarctica-1 | | JN294386 |
| subarctica-2 | | KU873996 |
| subarctica-3 | | KU873995 |
| ***Aeshna tuberculifera*** | |  |
| tuberculifera-1 | | HM413600 |
| tuberculifera-2 | | KR143160 |
| tuberculifera-3 | | KR141207 |
| ***Aeshan umbrosa*** | |  |
| umbrosa-1 | | MG470622 |
| umbrosa-2 | | MG466485 |
| umbrosa-3 | | MG466048 |
| ***Aeshna vercanica*** | |  |
| vercanica-1 | | KU180322 |
| vercanica-2 | | KU180303 |
| vercanica-3 | | KU180302 |
| ***Aeshna verticalis*** | |  |
| verticalis-1 | | JF839177 |
| verticalis-2 | | HM413555 |
| verticalis-3 | | HM413554 |
| ***Aeshna virdis*** | |  |
| viridis-1 | | KU180301 |
| viridis-2 | | KU180300 |
| ***Aeshna persephone*** | |  |
| persephone-1 | | KM50 |
| ***Anax junius -* Outgroup** | |  |
| AnaxJunius-1 | | KR143134.1 |
| AnaxJunius-2 | | KM536275.1 |
| AnaxJunius-3 | | KM531905.1 |
| ***Boyeria graffiana -* Outgroup** | |  |
| BoyeriaGraf-1 | | JN419358.1 |
| BoyeriaGraf-2 | | JN419357.1 |
| BoyeriaGraf-3 | | JN419356.1 |
| **Genus *Somatochlora*** | |  |
| ***Somatochlora albicincta*** | |  |
| AlbicinctaY1 | | MG847299 |
| AlbicinctaY2 | | MG847300 |
| AlbicinctaY3 | | MG847301 |
| AlbicinctaY4 | | MG847302 |
| AlbicinctaY5 | | MG847303 |
| AlbicinctaY6 | | MG847304 |
| AlbicinctaY7 | | MG847305 |
| AlbicinctaY8 | | MG847306 |
| AlbicinctaY9 | | MG847307 |
| AlbicinctaY10 | | MG847308 |
| AlbicinctaY11 | | MG847309 |
| AlbicinctaY13 | | MH560457 |
| AlbicinctaY14 | | MH560458 |
| AlbicinctaY15 | | MH560460 |
| AlbicinctaY16 | | MG847310 |
| AlbicinctaY17 | | MG847311 |
| AlbicinctaY18 | | MG847312 |
| AlbicinctaY19 | | MG847313 |
| ***Somatochlora alpestris*** | |  |
| Alpestris1 | | AB708912.1 |
| Alpestris2 | | AB708911.1 |
| Alpestris3 | | AB708910.1 |
| Alpestris4 | | AB708909.1 |
| Alpestris5 | | AB708908.1 |
| ***Somatochlora arctica*** | |  |
| Arctica1 | | AB708913.1 |
| ***Somatochlora clavata*** | |  |
| Clavata1 | | AB708916.1 |
| Clavata2 | | AB708914.1 |
| Clavata3 | | AB708915.1 |
| ***Somatochlora dido*** | |  |
| Dido1 | | AB708918.1 |
| Dido2 | | AB848397.1 |
| Dido3 | | AB848399.1 |
| Dido4 | | AB848398.1 |
| ***Somatochlora elongata*** | |  |
| Elongata1 | | JN420263.1 |
| Elongata2 | | JN420262.1 |
| Elongata3 | | KM528142.1 |
| Elongata4 | | JN420264.1 |
| ***Somatochlora exuberata*** | |  |
| Exuberata1 | | AB711468.1 |
| Exuberata2 | | AB711467.1 |
| Exuberata3 | | AB711466.1 |
| Exuberata4 | | AB708919.1 |
| Exuberata5 | | AB708924.1 |
| Exuberata6 | | AB708922.1 |
| Exuberata7 | | AB708923.1 |
| Exuberata8 | | AB708920.1 |
| Exuberata9 | | AB708921.1 |
| ***Somatochlora franklini*** | |  |
| Franklini1 | | KM534638.1 |
| Franklini2 | | KM531645.1 |
| FrankliniEP1 | | MG874111 |
| FrankliniEP2 | | MG874112 |
| FrankliniEP3 | | MG874113 |
| FrankliniEP4 | | MG874114 |
| FrankliniEP5 | | MG874115 |
| FrankliniEP6 | | MG874116 |
| FrankliniEP7 | | MG874117 |
| FrankliniEP8 | | MG874118 |
| FrankliniEP9 | | MG874119 |
| FrankliniEP10 | | MG874120 |
| FrankliniEP11 | | MG874121 |
| FrankliniEP12 | | MG874122 |
| FrankliniEP13 | | MG874123 |
| FrankliniEP14 | | MG874124 |
| ***Somatochlora graeseri*** | |  |
| Graeseri1 | | KF257081.1 |
| Graeseri2 | | AB708930.1 |
| Graeseri3 | | AB708928.1 |
| Graeseri4 | | AB708927.1 |
| Graeseri5 | | AB708926.1 |
| Graeseri6 | | AB708925.1 |
| Graeseri7 | | AB708929.1 |
| ***Somatochlora hudsonica*** | |  |
| HudsonicaEP1 | | MG874092 |
| HudsonicaEP2 | | MG874093 |
| HudsonicaEP3 | | MG874094 |
| HudsonicaEP4 | | MG874095 |
| HudsonicaEP5 | | MG874096 |
| HudsonicaEP6 | | MG874097 |
| HudsonicaEP7 | | MG874098 |
| HudsonicaEP8 | | MG874099 |
| HudsonicaEP9 | | MG874100 |
| HudsonicaEP10 | | MG874101 |
| HudsonicaEP11 | | MG874102 |
| HudsonicaEP12 | | MG874103 |
| HudsonicaEP13 | | MG874104 |
| HudsonicaEP14 | | MG874105 |
| HudsonicaEP15 | | MG874106 |
| HudsonicaEP16 | | MG874107 |
| HudsonicaEP17 | | MG874108 |
| HudsonicaEP18 | | MG874109 |
| HudsonicaEP19 | | MG874110 |
| ***Somatochlora metallica*** | |  |
| Metallica1 | | AB708932.1 |
| Metallica2 | | AB708931.1 |
| ***Somatochlora minor*** | |  |
| Minor1 | | JN420265.1 |
| ***Somatochlora sahlbergi*** | |  |
| SahlbergiEP1 | | MG874050 |
| SahlbergiEP2 | | MG874051 |
| SahlbergiEP3 | | MG874052 |
| SahlbergiEP4 | | MG874053 |
| SahlbergiEP5 | | MG874054 |
| SahlbergiEP6 | | MG874055 |
| SahlbergiEP7 | | MG874056 |
| SahlbergiEP8 | | MG874057 |
| SahlbergiEP9 | | MG874058 |
| SahlbergiEP10 | | MG874059 |
| SahlbergiEP11 | | MG874060 |
| SahlbergiEP12 | | MG874061 |
| SahlbergiEP13 | | MG874062 |
| SahlbergiEP14 | | MG874063 |
| SahlbergiEP15 | | MG874064 |
| SahlbergiEP16 | | MG874065 |
| SahlbergiEP17 | | MG874066 |
| SahlbergiEP18 | | MG874067 |
| SahlbergiEP19 | | MG874068 |
| SahlbergiEP20 | | MG874069 |
| SahlbergiEU1_Norway | | MG847280 |
| SahlbergiEU2_Norway | | MG847281 |
| SahlbergiEU3_Norway | | MG847282 |
| SahlbergiEU4_Sweden | | MG847283 |
| SahlbergiEU5_Finland | | MG847284 |
| SahlbergiEU6_Norway | | MG847285 |
| SahlbergiEU7_Norway | | MH560456 |
| SahlbergiEU8_Norway | | MG847286 |
| SahlbergiEU9_Sweden | | MG847287 |
| SahlbergiEU10_Norway | | MG847288 |
| SahlbergiEU11_Sweden | | MG847289 |
| SahlbergiEU12_Finland | | MG847290 |
| SahlbergiEU13_Finland | | MG847291 |
| SahlbergiEU14_Norway | | MG847292 |
| SahlbergiEU15_Norway | |  |
| SahlbergiEU16_Norway | | MG847293 |
| SahlbergiEU17_Norway | | MG847294 |
| SahlbergiEU18_Norway | | MG847295 |
| SahlbergiEU19_Norway | | MG847296 |
| SahlbergiEU20_Norway | | MG847297 |
| SahlbergiEU21_Norway | | MG847298 |
| SahlbergiEU22_Finland | | MH560459 |
| SahlbergiNA1 | | MG847274 |
| SahlbergiNA2 | | MG847275 |
| SahlbergiNA3 | | MG847276 |
| SahlbergiNA4 | | MG847277 |
| SahlbergiNA5 | | MG847278 |
| SahlbergiNA6 | | MG847279 |
| ***Somatochlora semicircularis*** | |  |
| Semicicularis1 | | KM529041.1 |
| ***Somatochlora septentrionalis*** | |  |
| SeptrentionalisEP2 | | MG874076 |
| SeptrentionalisEP3 | | MG874077 |
| SeptrentionalisEP4 | | MG874078 |
| SeptrentionalisEP5 | | MG874079 |
| SeptrentionalisEP6 | | MG874080 |
| SeptrentionalisEP7 | | MG874081 |
| SeptrentionalisEP8 | | MG874082 |
| SeptrentionalisEP9 | | MG874083 |
| SeptrentionalisEP10 | | MG874084 |
| SeptrentionalisEP11 | | MG874085 |
| SeptrentionalisEP12 | | MG874086 |
| SeptrentionalisEP13 | | MG874087 |
| SeptrentionalisEP14 | | MG874088 |
| SeptrentionalisEP15 | | MG874089 |
| SeptrentionalisEP16 | | MG874090 |
| SeptrentionalisEP17 | | MG874091 |
| ***Somatochlora uchidai*** | |  |
| Uchidai1 | | AB708936.1 |
| Uchidai2 | | AB708935.1 |
| Uchidai3 | | AB708933.1 |
| Uchidai4 | | AB708934.1 |
| ***Somatochlora viridiaenea*** | |  |
| Viridiaenea1 | | AB708938.1 |
| Viridiaenea2 | | AB708937.1 |
| ***Somatochlora whitehousei*** | |  |
| WhitehouseiEP1 | | MG874070 |
| WhitehouseiEP2 | | MG874071 |
| WhitehouseiEP3 | | MG874072 |
| WhitehouseiEP4 | | MG874073 |
| WhitehouseiEP5 | | MG874074 |
| WhitehouseiEP6 | | MG874075 |
| ***Somatochlora williamsoni*** | |  |
| Williamsoni1 | | KM531663.1 |
| Somatochlora sp. | |  |
| SomatSP1 | | JN420261.1 |
| SomatSP2 | | JN420260.1 |
| SomatSP3 | | JN420259.1 |
| SomatSP4 | | JN420250.1 |
| SomatSP5 | | JN420256.1 |
| SomatSP6 | | JN420244.1 |
| SomatSP7 | | JN420242.1 |
| SomatSP8 | | JN420236.1 |
| SomatSP9 | | JN420232.1 |
| SomatSP10 | | JN420234.1 |
| SomatSP11 | | JN420240.1 |
| SomatSP12 | | JN420245.1 |
| SomatSP13 | | JN420249.1 |
| SomatSP14 | | JN420251.1 |
| SomatSP15 | | JN420258.1 |
| ***Cordulia amurensis*** | |  |
| CordAmu | | AB708895.1 |
| ***Helocorculia uhleri* Outgroup** | |  |
| Uhleri1 | |  |
| Uhleri2 | |  |
| ***Genus Libellula*** | |  |
| ***Libellula angelina*** | |  |
| angelina-1 | | AF195733.1 |
| ***Libellula auripennis*** | |  |
| auripennis-1 | | AF195734.1 |
| ***Libellula axilena*** | |  |
| axilena-1 | | AF195735.1 |
| ***Libellula comanche*** | |  |
| comanche-1 | | AF195736.1 |
| ***Libellula composita*** | |  |
| composita-1 | | AF195737.1 |
| ***Libellula croceipennis*** | |  |
| croceipennis-1 | | AF195738.1 |
| ***Libellula cyanea*** | |  |
| cyanea-1 | | AF195739.1 |
| **Libellula flavida** | |  |
| flavida-1 | | AF195743.1 |
| ***Libellula forensis*** | |  |
| forensis-1 | | AF195744.1 |
| ***Libellula incesta*** | |  |
| incesta-1 | | AF195746.1 |
| ***Libellula jesseana*** | |  |
| jesseana-1 | | AF195747.1 |
| ***Libellula luctuosa*** | |  |
| luctuosa-1 | | AF195749.1 |
| ***Libellula needhami*** | |  |
| needhami-1 | | AF195751.1 |
| ***Libellula nodostica*** | |  |
| nodostica-1 | | AF195752.1 |
| ***Libellula pulchella*** | |  |
| pulchella-1 | | AF195753.1 |
| ***Libellula quadrimaculata*** | |  |
| quadrimaculata-1 | | AF195758.1 |
| ***Ladona julia Outgroup*** | |  |
| Ladona-1 | | AF195748.1 |
| ***Crocothemis servilia* Outgroup** | | |
| Crocothem-2 | MF774561.1 | |
| **Genus *Sympetrum*** |  | |
| ***Sympetrum ambiguum*** |  | |
| ambiguum-1 | EF636300.1 | |
| *Sympetrum bacca* |  | |
| bacca-1 | AB709114.1 | |
| bacca-2 | AB709115.1 | |
| bacca-3 | LC366695.1 | |
| bacca-4 | LC366696.1 | |
| bacca-5 | KF257091.1 | |
| ***Sympetrum cordulegaster*** |  | |
| cordulegaster-1 | AB709116.1 | |
| ***Sympetrum corruptum*** |  | |
| corruptum-1 | KM529511.1 | |
| corruptum-2 | JN294353.1 | |
| corruptum-3 | JN294379.1 | |
| ***Sympetrum costiferum*** |  | |
| costiferum-1 | EF636248.1 | |
| costiferum-2 | EF636249.1 | |
| costiferum-3 | EF636250.1 | |
| costiferum-4 | KM529127.1 | |
| costiferum-5 | KR141601.1 | |
| costiferum-6 | HM413468.1 | |
| costiferum-7 | JN294511.1 | |
| costiferum-8 | JN294515.1 | |
| costiferum-9 | JN294428.1 | |
| ***Sympetrum croceolum*** |  | |
| croceolum-1 | JQ772574.1 | |
| croceolum-2 | AB709117.1 | |
| croceolum-3 | AB709118.1 | |
| croceolum-4 | AB709119.1 | |
| croceolum-5 | LC366659.1 | |
| ***Sympetrum danaeA*** |  | |
| danaeA-1 | JN294419 | |
| danaeA-2 | JN294425 | |
| danaeA-3 | JN294426 | |
| danaeA-4 | JN294429 | |
| danaeA-5 | JN294355 | |
| danaeA-6 | JN294359 | |
| danaeA-7 | JN294378 | |
| danaeA-8 | BritC-20 | |
| danaeA-9 | KM532431 | |
| danaeA-10 | KM531697 | |
| danaeA-11 | KM528837 | |
| danaeA-12 | KM529124 | |
| danaeA-13 | EU243819 | |
| danaeA-14 | EU243818 | |
| danaeA-15 | EU243853 | |
| danaeA-16 | EU243823 | |
| danaeA-17 | EU243821 | |
| danaeA-18 | EU243817 | |
| danaeA-19 | EU243869 | |
| ***Sympetrum danaeB*** |  | |
| danaeB-1 | MN847808 | |
| danaeB-2 | MN847809 | |
| danaeB-3 | MN847810 | |
| danaeB-4 | MN847811 | |
| danaeB-5 | MN847797 | |
| danaeB-6 | MN847812 | |
| danaeB-7 | MN847815 | |
| danaeB-8 | MN847816 | |
| danaeB-9 | MN847817 | |
| ***Sympetrum darwinianum*** |  | |
| darwinianum-1 | JQ772575.1 | |
| darwinianum-2 | AB709123.1 | |
| darwinianum-3 | AB709124.1 | |
| darwinianum-4 | LC366674.1 | |
| darwinianum-5 | LC366693.1 | |
| darwinianum-6 | LC366869.1 | |
| darwinianum-7 | KF257097.1 | |
| ***Sympetrum depressiusculum*** |  | |
| depressiusculum-1 | JQ772576.1 | |
| depressiusculum-2 | AB709125.1 | |
| depressiusculum-3 | AB709126.1 | |
| depressiusculum-4 | AB709127.1 | |
| depressiusculum-5 | AB709139.1 | |
| depressiusculum-6 | AB709140.1 | |
| ***Sympetrum eroticum*** |  | |
| eroticum-1 | JQ772577.1 | |
| eroticum-2 | AB709145.1 | |
| eroticum-3 | AB709146.1 | |
| eroticum-4 | AB709147.1 | |
| eroticum-5 | LC366675.1 | |
| eroticum-6 | KF257077.1 | |
| ***Sympetrum flaveolum*** |  | |
| flaveolum-1 | JQ772578.1 | |
| flaveolum-2 | AB709154.1 | |
| flaveolum-3 | AB709155.1 | |
| ***Sympetrum fonscolombii*** |  | |
| fonscolombii-1 | JQ772579.1 | |
| fonscolombii-2 | MF774497.1 | |
| fonscolombii-3 | AB709156.1 | |
| fonscolombii-4 | AB709157.1 | |
| fonscolombii-5 | LC366734.1 | |
| fonscolombii-6 | KF257098.1 | |
| ***Sympetrum frequens*** |  | |
| frequens-1 | AB709158.1 | |
| frequens-2 | AB709159.1 | |
| frequens-3 | AB709160.1 | |
| frequens-4 | LC366661.1 | |
| frequens-5 | KF257076.1 | |
| ***Sympetrum gracile*** |  | |
| gracile-1 | JQ772580.1 | |
| gracile-2 | AB709166.1 | |
| gracile-3 | AB709167.1 | |
| ***Sympetrum illotum*** |  | |
| illotum-1 | JQ772581.1 | |
| ***Sympetrum infuscatum*** |  | |
| infuscatum-1 | JQ772582.1 | |
| infuscatum-2 | AB709168.1 | |
| infuscatum-3 | AB709169.1 | |
| infuscatum-4 | LC366672.1 | |
| infuscatum-5 | LC366694.1 | |
| infuscatum-6 | LC366870.1 | |
| infuscatum-7 | KF257078.1 | |
| ***Sympetrum internum*** |  | |
| internum-1 | JQ772583.1 | |
| internum-2 | EF636303.1 | |
| internum-3 | EF636304.1 | |
| internum-4 | EF636302.1 | |
| internum-5 | EF636305.1 | |
| internum-6 | KM537207.1 | |
| internum-7 | KM528570.1 | |
| internum-8 | KM529618.1 | |
| internum-9 | KM528498.1 | |
| internum-10 | KM535304.1 | |
| internum-11 | KR146063.1 | |
| internum-12 | KU876275.1 | |
| internum-13 | KU876274.1 | |
| ***Sympetrum janeae*** |  | |
| janeae-1 | EF636310.1 | |
| janeae-2 | EF636311.1 | |
| janeae-3 | KM537594.1 | |
| janeae-4 | KM533802.1 | |
| janeae-5 | KM537115.1 | |
| ***Sympetrum kunckeli*** |  | |
| kunckeli-1 | JQ772584.1 | |
| kunckeli-2 | AB709170.1 | |
| kunckeli-3 | AB709171.1 | |
| kunckeli-4 | KF257092.1 | |
| ***Sympetrum maculatum*** |  | |
| maculatum-1 | JQ772585.1 | |
| maculatum-2 | AB709172.1 | |
| maculatum-3 | AB709173.1 | |
| maculatum-4 | AB709174.1 | |
| ***Sympetrum madidum*** |  | |
| madidum-1 | JQ772586.1 | |
| madidum-2 | KM529557.1 | |
| madidum-3 | KM532060.1 | |
| madidum-4 | JN294351.1 | |
| ***Sympetrum meridionale*** |  | |
| meridionale-1 | JQ772587.1 | |
| meridionale-2 | EF636213.1 | |
| meridionale-3 | EF636214.1 | |
| ***Sympetrum nigrecens*** |  | |
| nigrecens-1 | EF636215.1 | |
| nigrecens-2 | EF636216.1 | |
| nigrecens-3 | EF636217.1 | |
| nigrecens-4 | EF636218.1 | |
| ***Sympetrum obstrusum*** |  | |
| obstrusum-1 | EF636319.1 | |
| obstrusum-2 | EF636320.1 | |
| obstrusum-3 | EF636321.1 | |
| obstrusum-4 | KM534092.1 | |
| obstrusum-5 | KM529313.1 | |
| obstrusum-6 | KM535245.1 | |
| obstrusum-7 | KX781839.1 | |
| obstrusum-8 | KX781917.1 | |
| ***Sympetrum occidentale*** |  | |
| occidentale-1 | EF636251.1 | |
| occidentale-2 | EF636252.1 | |
| occidentale-3 | EF636263.1 | |
| occidentale-4 | EF636264.1 | |
| occidentale-5 | EF636266.1 | |
| occidentale-6 | EF636267.1 | |
| occidentale-7 | EF636268.1 | |
| occidentale-8 | EF636269.1 | |
| ***Sympetrum pallipes*** |  | |
| pallipes-1 | EF636329.1 | |
| pallipes-2 | KM536273.1 | |
| pallipes-3 | KM534409.1 | |
| **Sympetrum parvulum** |  | |
| parvulum-1 | DQ279743.1 | |
| parvulum-2 | JQ772588.1 | |
| parvulum-3 | AB709177.1 | |
| parvulum-4 | LC366871.1 | |
| parvulum-5 | KF257088.1 | |
| ***Sympetrum pedemontanum*** |  | |
| pedemontanum-1 | JQ772589.1 | |
| pedemontanum-2 | AB709179.1 | |
| pedemontanum-3 | AB709180.1 | |
| pedemontanum-4 | KF257095.1 | |
| ***Sympetrum risi*** |  | |
| risi-1 | JQ772590.1 | |
| risi-2 | AB709183.1 | |
| risi-3 | AB709184.1 | |
| risi-4 | LC366673.1 | |
| risi-5 | LC366702.1 | |
| risi-6 | KF257127.1 | |
| ***Sympetrum rubicundulum*** |  | |
| rubicundulum-1 | EF636331.1 | |
| rubicundulum-2 | EF636332.1 | |
| rubicundulum-3 | EF636333.1 | |
| rubicundulum-4 | EF636330.1 | |
| ***Sympetrum sanguineum*** |  | |
| sanguineum-1 | EF636237.1 | |
| ***Sympetrum signiferum*** |  | |
| signiferum-1 | EF636280.1 | |
| signiferum-2 | EF636281.1 | |
| signiferum-3 | EF636287.1 | |
| signiferum-4 | EF636288.1 | |
| ***Sympetrum speciosum*** |  | |
| speciosum-1 | JQ772591.1 | |
| speciosum-2 | AB709187.1 | |
| speciosum-3 | AB709189.1 | |
| speciosum-4 | LC366678.1 | |
| speciosum-5 | LC366760.1 | |
| speciosum-6 | KF257089.1 | |
| ***Sympetrum striolatum*** |  | |
| striolatum-1 | EF636220.1 | |
| striolatum-2 | EF636236.1 | |
| striolatum-3 | EF636238.1 | |
| striolatum-4 | EF636245.1 | |
| striolatum-5 | AB709190.1 | |
| striolatum-6 | LC366663.1 | |
| striolatum-7 | KF257086.1 | |
| ***Sympetrum uniforme*** |  | |
| uniforme-1 | JQ772592.1 | |
| uniforme-2 | AB709192.1 | |
| uniforme-3 | LC366900.1 | |
| uniforme-4 | KF257087.1 | |
| ***Sympetrum vicinum*** |  | |
| vicinum-1 | EF636289.1 | |
| vicinum-2 | EF636290.1 | |
| vicinum-3 | EF636299.1 | |
| vicinum-4 | HM413479.1 | |
| ***Sympetrum villosum*** |  | |
| villosum-1 | JQ772593.1 | |
| ***Sympetrum vulgatum*** |  | |
| vulgatum-1 | EF636246.1 | |
| vulgatum-2 | AB709195.1 | |
| vulgatum-3 | LT898333.1 | |
| vulgatum-4 | LT634113.1 | |
| ***Celithemis eponina*** |  | |
| CelithEP-1 | MG375029.1 | |
| ***Celithemis elisa*** |  | |
| CelithE-1 | KM531025.1 | |
| ***Leucorrhinia proxima*** |  | |
| LeucoP-1 | JF839329.1 | |
| LeucoP-2 | JF839328.1 | |
| ***Leucorrhinia borealis*** |  | |
| LeucoB-1 | JF839327.1 | |
| LeucoB-2 | JF839326.1 | |

**Table S3.2.** **List of models for different partitions.**

| Alignment | Partitions | Iqtree model based on BIC (Kalyaanamoorthy et al 2017) |
| --- | --- | --- |
| *Aeshna* | 1 | TNe{5.6533,100}+FQ+G4{0.191129} |
|  | 2 | F81+F |
|  | 3 | TPM2u{0.142618,8.84618}+F{0.521441,0.047049,0.0359156,0.395594}+G4{0.743497} |
| *Aeshna juncea* | 1 | TNe{1.73253,23.8861}+FQ+I{0.577239} |
|  | 2 | F81+F |
|  | 3 | TPM2{0.140674,6.53772}+F{0.525822,0.0565816,0.0426242,0.374973}+G4{0.676001} |
| *Aeshna subarctica* | 1 | K2P{100}+FQ |
|  | 2 | F81 |
|  | 3 | HKY{100}+F{0.530316,0.0500651,0.0405246,0.379094} |
| *Sympetrum* | 1 | TIM{0.969605,0.28767,36.0424}+F{0.278179,0.175338,0.317118,0.229366}+I{0.653662}+G4{1.24674} |
|  | 2 | F81+F |
|  | 3 | TIM{14.8798,0.437036,9.44335}+F{0.442198,0.109281,0.0627222,0.385799}+I{0.0373664}+G4{1.64774} |
| *Sympetrum danae* | 1 | TN{0.0001,44.3573}+F{0.271611,0.154898,0.329671,0.24382} |
|  | 2 | F81+F |
|  | 3 | K3Pu{13.1973,0.0753976}+F{0.441158,0.0914249,0.071959,0.395458}+G4{0.717922} |
| *Libellula* | 1 | TIM2e{3.76625,4.08781,80.5571}+FQ+I{0.500691} |
|  | 2 | TN{2.225,0.220111}+F{0.154887,0.264767,0.158286,0.422061} |
|  | 3 | HKY{41.0997}+F{0.48694,0.0891112,0.0406785,0.383271}+G4{0.9449} |
| *Libellula quadrimaculata* | 1 | TNe{7.27166,100}+FQ |
|  | 2 | F81+F |
|  | 3 | TN{40.6664,14.7249}+F{0.483984,0.060961,0.021021,0.434034}+G4{1.21954} |
| *Somatochlora* | 1 | TIM2e{5.42461,5.31518,97.3475}+FQ+G4{0.209356} |
|  | 2 | F81+F |
|  | 3 | TIM{18.5205,0.430129,8.54267}+F{0.514637,0.109515,0.0225772,0.353271}+G4{0.980017} |
| *Somatochlora sahlbergi* | 1 | JC |
|  | 2 | F81+F |
|  | 3 | TPM3{48.2029,100}+F{0.506398,0.0944549,0.0207195,0.378428} |

**Table S3.3.** **List of priors and fossils used.**

| Genus/Species |  | Priors | | Prior Shape |
| --- | --- | --- | --- | --- |
|  | Fossil species | Lower | Upper |  |
| Genus *Aeshna* | *Aeshna shanwangensis* (Li et al 2011) | 16 My | 176.7 My^a^ | Uniform distribution |
| Genus *Sympetrum* | *Sympetrum bigoti* (Nel et 1994) | 23 My | 90.8 My^a^ | Uniform distribution |
| Genus *Libellula* | *Libellula requieniana* (Nicolas 1890) | 23 My | 90.8 My^a^ | Uniform distribution |
| Genus *Somatochlora* | *Somatochlora oregonica* (Cockerell 1927) | 33 My | 61.04 My^a^ | Uniform distribution |
|  |  |  |  |  |
| *Aeshna juncea* |  | 220 Ky^b^ | 6.9 My^c^ | Uniform distribution |
| *Aeshna subarctica* |  | 220 Ky^b^ | 6.9 My^c^ | Uniform distribution |
| *Sympetrum danae* (grp A*)* |  | 120 Ky^b^ | 4.1 My^c^ | Uniform distribution |
| *Sympetrum danae* (grp B) |  | 120 Ky^b^ | 4.1 My^c^ | Uniform distribution |
| *Libellula quadrimaculata* |  | 565 Ky^b^ | 5.63 My^c^ | Uniform distribution |
| *Somatochlora sahlbergi* |  | 200 Ky^b^ | 2.92 My^c^ | Uniform distribution |

^a^ Maximum age for families, Aeshnidae, Cordullidae and Libellulidae estimated based on paleoptera transcriptomes (Kohli et al., in prep) were used as the maximum of the genera *Aeshna* (Aeshnidae), *Somatochlora* (Cordulidae), *Libellula* (Libellulidae) and *Sympetrum* (Libellulidae). The oldest know fossil ages for the families Aeshnidae, Cordulidae and Libellulidae are 138.9 mya, 12.7mya and 29.2mya. Therefore we choose to use estimates from divergence time estimation analysis (from Kohli et al (in prep)) since they are older of the two.

^b^ Minimum age for the species recovered in the divergence time estimation of the genus that it belongs to.

^c^ Maximum age for the species recovered in the divergence time estimation of the genus that it belongs to.

Table S4. Polymorphism statistics for the five species of Holarctic dragonflies. S, number of segregating sites, pairwise nucleotide diversity (π) and number of haplotypes (h).

| *Aeshna juncea* | Sample size | S | Pi (π) | h |
| --- | --- | --- | --- | --- |
| North America | 33 | 16 | 2.76136 | 8 |
| China | 2 | 16 | 16 | 2 |
| Europe | 31 | 17 | 2.67527 | 10 |
| Japan | 8 | 25 | 12.39286 | 5 |
| Russia | 16 | 24 | 8.625 | 8 |
| Yukon/ Alaska | 16 | 20 | 4.84167 | 6 |
| Mean | 17.66667 | 19.66667 | 7.88269 |  |
| s.d. | 12.30718 | 4.0332 | 5.46583 |  |
|  |  |  |  |  |
| *Aeshna subarctica* | Sample size | S | Pi (π) | h |
| North America | 4 | 3 | 1.5 | 3 |
| Europe | 20 | 4 | 1.67368 | 3 |
| Japan | 4 | 0 | 0 | 1 |
| Yukon/Alaska | 5 | 1 | 0.4 | 2 |
| Mean | 8.25 | 2 | 0.89342 |  |
| s.d. | 7.8475 | 1.82574 | 0.82025 |  |
|  |  |  |  |  |
| *Libellula quadrimaculata* | Sample size | S | Pi (π) | h |
| North America | 34 | 27 | 2.85383 | 17 |
| China | 2 | 12 | 12 | 2 |
| Europe | 17 | 4 | 0.47059 | 5 |
| Japan | 3 | 5 | 3.33333 | 3 |
| Yukon/Alaska | 4 | 6 | 3.16667 | 4 |
| Mean | 12 | 10.8 | 4.36488 |  |
| s.d. | 13.72953 | 9.57601 | 4.42277 |  |
|  |  |  |  |  |
| *Sympetrum danae*-A | Sample size | S | Pi (π) | h |
| YT/AK | 6 | 0 | 0 | 1 |
| CA | 48 | 6 | 0.36436 | 6 |
| NA | 34 | 8 | 1.34759 | 4 |
| Mean | 29.33333 | 4.66667 | 0.57065 |  |
| s.d. | 21.38535 | 4.16333 | 0.69708 |  |
|  |  |  |  |  |
| *Sympetrum danae*-B | Sample size | S | Pi (π) | h |
| YT/AK | 21 | 5 | 0.9619 | 5 |
| Europe | 33 | 5 | 0.35985 | 4 |
| North America (Canada) | 2 | 1 | 1 | 2 |
| Russia | 12 | 3 | 0.77273 | 3 |
| Japan | 3 | 3 | 2 | 2 |
| Mean | 14.2 | 3.4 | 1.0189 |  |
| s.d. | 13.0269 | 1.67332 | 0.60438 |  |


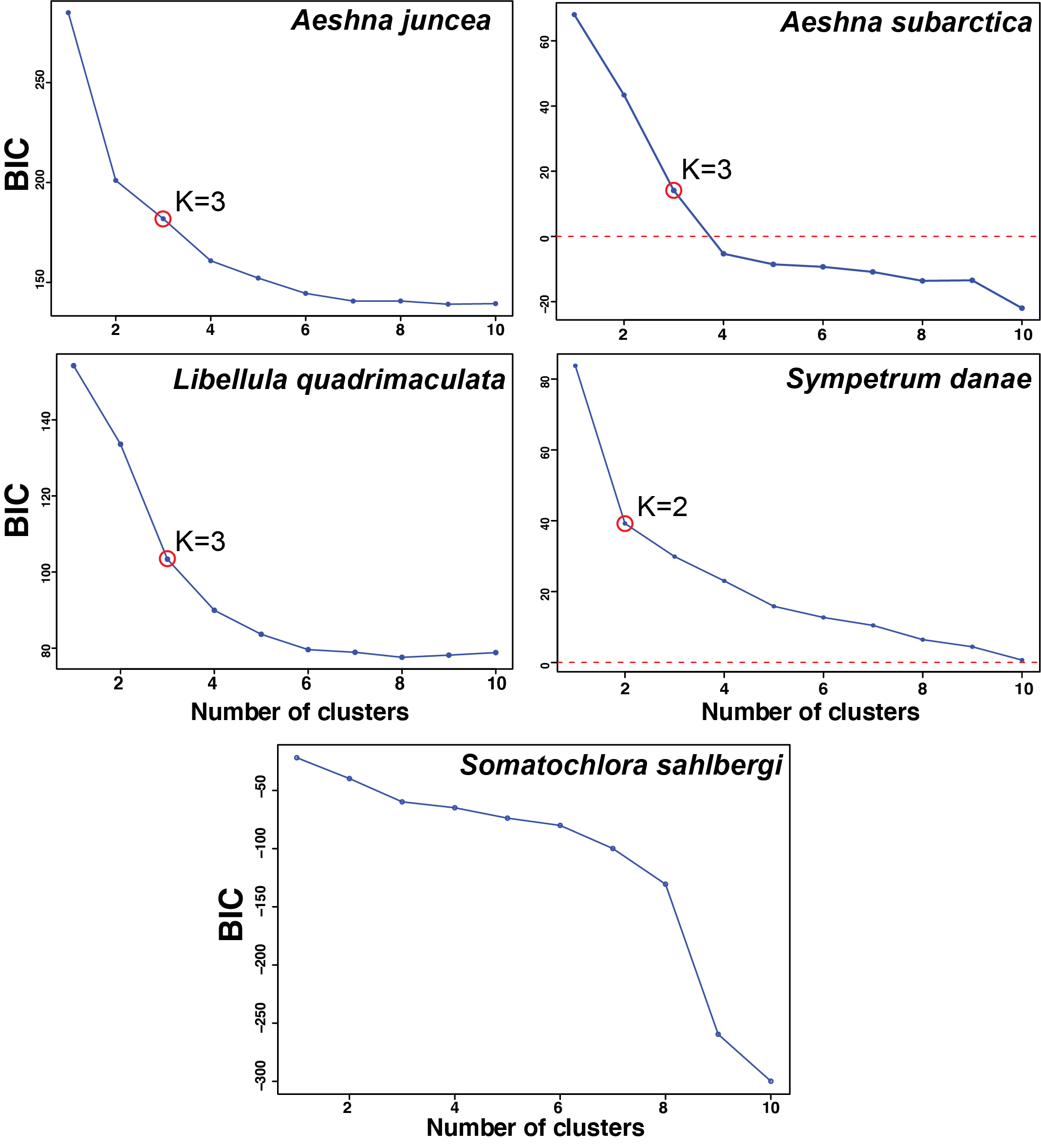


S1 Figure. **Value of BIC versus number of clusters.** Red circle indicates the value of cluster chosen for each species.


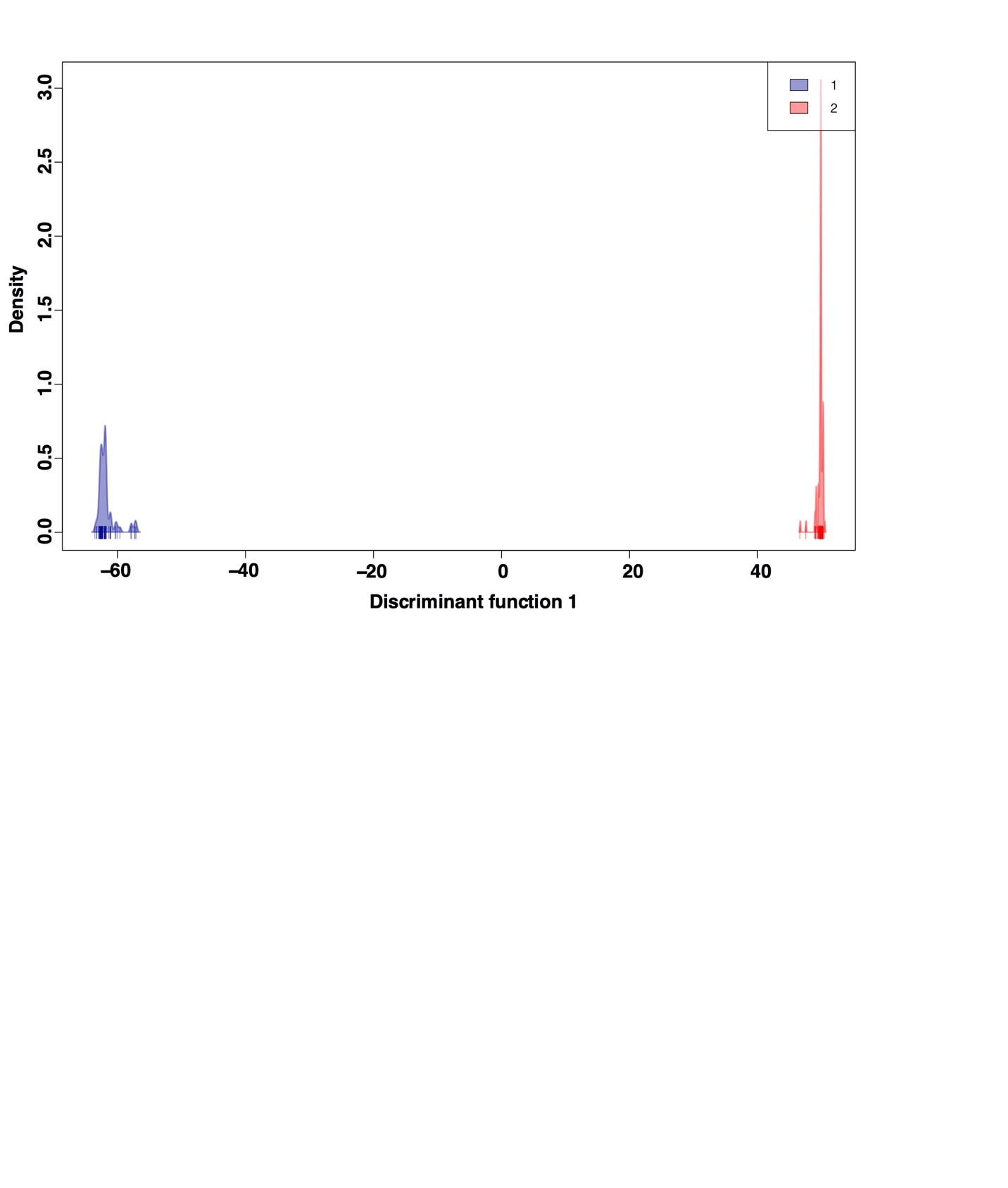


S2 Figure. **Density plot along Discriminant function 1** for all samples of S. danae, showing differentiation among Group A and B.

**References Cites in Supplementary Information**

Cockerell, T. D. A. (1927). Tertiary Fossil Insects from Eastern Oregon. In R. Kellogg, J. Merriam, C. Stock, R. Chaney, H. Mason (eds.), *Additions to the Paleontology of the Pacific Coast and Great Basin Regions of North America* **346**:64-65

Kalyaanamoorthy, S., Minh, B. Q., Wong, T. K., von Haeseler, A., & Jermiin, L. S. (2017). ModelFinder: fast model selection for accurate phylogenetic estimates. *Nature methods*, *14*(6), 587.

Li, Y. J., Nel, A., Ren, D., Zhang, B. L., & Pang, H., (2011). New discoveries of Neogene hawker dragonflies (Insecta, Odonata, Aeshnidae) from Shandong province in China. *Zoosystema* **33**:577-590

Nel, A., Martínez-Delclòs, X., Escuillé, F., & Brisac, P. (1994). Les Aeshnidae fossiles: Etat actuel des connaissances (Odonata, Anisoptera). *Neues Jahrbuch für Geologie und Paläontologie, Abhandlungen* **194(2/3)**:143-186

Nicolas, H. (1890). Insectes fossiles d'Aix - collection du Muséum Requien, à Avignon. *Compte Rendus, Association Française pour l'Avancement des Sciences* **18**:424-432

Rambaut A, Drummond AJ, Xie D, Baele G and Suchard MA (2018) Posterior summarisation in Bayesian phylogenetics using Tracer 1.7. Systematic Biology. **syy032**.

Simonsen, T. J., Olsen, K., & Djernæs, M. (in review). The African-Iberian connection in Odonata: mtDNA and ncDNA based phylogeography of *Aeshna cyanea* (Müller) (Odonata: Aeshnidae) in Western Palaearctic. Submitted to *Arthropod Systematics and Phylogeny*

Suchard MA, Lemey P, Baele G, Ayres DL, Drummond AJ, Rambaut A. (2018) Bayesian phylogenetic and phylodynamic data integration using BEAST 1.10. *Virus evolution*. 4(1)16.

Trifinopoulos, J., Nguyen, L. T., von Haeseler, A., & Minh, B. Q. (2016). W-IQ-TREE: a fast online phylogenetic tool for maximum likelihood analysis. *Nucleic acids research*, *44*(W1), W232-W235.
